# Tobacco Product Use and Associated Factors Among Middle and High School Students —  United States, 2019

**DOI:** 10.15585/mmwr.ss6812a1

**Published:** 2019-11-06

**Authors:** Teresa W. Wang, Andrea S. Gentzke, MeLisa R. Creamer, Karen A. Cullen, Enver Holder-Hayes, Michael D. Sawdey, Gabriella M. Anic, David B. Portnoy, Sean Hu, David M. Homa, Ahmed Jamal, Linda J. Neff

**Affiliations:** 1 Office on Smoking and Health, National Center for Chronic Disease Prevention and Health Promotion, CDC; 2 Center for Tobacco Products, Food and Drug Administration, Silver Spring, Maryland

## Abstract

**Problem/Condition:**

Tobacco use is the leading cause of preventable disease, disability, and death in the United States. Most tobacco product use begins during adolescence. In recent years, tobacco products have evolved to include various smoked, smokeless, and electronic products.

**Period Covered:**

2019.

**Description of System:**

The National Youth Tobacco Survey (NYTS) is an annual, cross-sectional, school-based, self-administered survey of U.S. middle school (grades 6–8) and high school (grades 9–12) students. A three-stage cluster sampling procedure is used to generate a nationally representative sample of U.S. students attending public and private schools. NYTS is the only nationally representative survey of U.S. middle and high school students that focuses exclusively on tobacco use patterns and associated factors. NYTS is designed to provide national data on tobacco product use and has been conducted periodically during 1999–2009 and annually since 2011. Data from NYTS are used to support the design, implementation, and evaluation of comprehensive tobacco use prevention and control programs and to inform tobacco regulatory activities. Since its inception in 1999 through 2018, NYTS had been conducted via paper and pencil questionnaires. In 2019, NYTS for the first time was administered in schools using electronic data collection methods. CDC’s Office on Smoking and Health, in collaboration with the U.S. Food and Drug Administration’s (FDA’s) Center for Tobacco Products, analyzed data from the 2019 NYTS to assess tobacco product use patterns and associated factors among U.S. middle and high school students. Overall, 19,018 questionnaires were completed and weighted to represent approximately 27.0 million students. On the basis of self-reported grade level, this included 8,837 middle school questionnaires (11.9 million students) and 10,097 high school questionnaires (15.0 million students); 84 questionnaires with missing information on grade level were excluded from school-level analyses.

**Results:**

In 2019, an estimated 53.3% of high school students (8.0 million) and 24.3% of middle school students (2.9 million) reported having ever tried a tobacco product. Current (past 30-day) use of a tobacco product (i.e., electronic cigarettes [e-cigarettes], cigarettes, cigars, smokeless tobacco, hookahs, pipe tobacco, and bidis [small brown cigarettes wrapped in a leaf]) was reported by 31.2% of high school students (4.7 million) and 12.5% of middle school students (1.5 million). E-cigarettes were the most commonly cited tobacco product currently used by 27.5% of high school students (4.1 million) and 10.5% of middle school students (1.2 million), followed in order by cigars, cigarettes, smokeless tobacco, hookahs, and pipe tobacco. Tobacco product use also varied by sex and race/ethnicity. Among current users of each tobacco product, the prevalence of frequent tobacco product use (on ≥20 days of the preceding 30 days) ranged from 16.8% of cigar smokers to 34.1% of smokeless tobacco product users. Among current users of each individual tobacco product, e-cigarettes were the most commonly used flavored tobacco product (68.8% of current e-cigarette users). Among students who reported ever having tried e-cigarettes, the three most commonly selected reasons for use were “I was curious about them” (55.3%), “friend or family member used them” (30.8%), and “they are available in flavors, such as mint, candy, fruit, or chocolate” (22.4%). Among never users of each individual tobacco product, curiosity and susceptibility (a construct that can help to identify future tobacco product experimentation or use) was highest for e-cigarettes (39.1% and 45.0%, respectively) and cigarettes (37.0% and 45.9%, respectively). Overall, 86.3% of students who reported contact with an assessed potential source of tobacco product advertisements or promotions (going to a convenience store, supermarket, or gas station; using the Internet; watching television or streaming services or going to the movies; or reading newspapers or magazines) reported exposure to marketing for any tobacco product; 69.3% reported exposure to e-cigarette marketing and 81.7% reported exposure to marketing for cigarettes or other tobacco products. Among all students, perceiving no harm or little harm from intermittent tobacco product use (use on some days but not every day) was 28.2% for e-cigarettes, 16.4% for hookahs, 11.5% for smokeless tobacco products, and 9.5% for cigarettes. Among current users of any tobacco product, 24.7% reported experiencing cravings to use tobacco products during the past 30 days and 13.7% reported wanting to use a tobacco product within 30 minutes of waking. Moreover, 57.8% of current tobacco product users reported they were seriously thinking about quitting the use of all tobacco products and 57.5% reported they had stopped using all tobacco products for ≥1 day because they were trying to quit.

**Interpretation:**

In 2019, approximately one in four youths (23.0%) had used a tobacco product during the past 30 days. By school level, this represented approximately three in 10 high school students (31.2%) and approximately one in eight middle school students (12.5%). Since 2014, e-cigarettes have been the most commonly used tobacco product among youths. Importantly, more than half of current youth tobacco product users reported seriously thinking about quitting all tobacco products in 2019. However, established factors of use and initiation, including the availability of flavors, exposure to tobacco product marketing, curiosity and susceptibility, and misperceptions about harm from tobacco product use, remained prevalent in 2019 and continue to promote tobacco product use among youths.

**Public Health Action:**

The continued monitoring of all forms of youth tobacco product use and associated factors through surveillance efforts including NYTS is important to the development of public health policy and action at national, state, and community levels. Everyone, including public health professionals, health care providers, policymakers, educators, parents, and others who influence youths, can help protect youths from the harms of all tobacco products. In addition, the comprehensive and sustained implementation of evidence-based tobacco control strategies, combined with FDA’s regulation of tobacco products, is important for reducing all forms of tobacco product use among U.S. youths.

## Introduction

Tobacco product use is the leading cause of preventable disease, disability, and death in the United States (*1*). Preventing tobacco product use among youths is critical to decreasing morbidity and mortality because nearly all tobacco product use begins during youth or young adulthood; approximately nine in 10 adult cigarette smokers start before age 18 years (*1*–*3*). In recent years, tobacco products have evolved to include various smoked, smokeless, and electronic products.

The National Youth Tobacco Survey (NYTS), conducted periodically during 1999–2009 and annually since 2011, provides national data on estimates of tobacco product use to support the design, implementation, and evaluation of comprehensive youth tobacco prevention and control programs and to inform tobacco regulatory activities in the United States (*4*). NYTS is the only nationally representative survey of U.S. middle school (grades 6–8) and high school (grades 9–12) students that focuses exclusively on tobacco product use and associated factors.

This report uses findings from the 2019 NYTS to describe the prevalence of youth tobacco product use and selected associated factors, including flavored tobacco product use, reasons for use, exposure to tobacco product marketing, curiosity and susceptibility, harm perceptions, urges to use tobacco products, and quitting behaviors. These findings can be used by public health professionals, health care providers, policymakers, educators, parents, and others who influence youths to prevent and reduce tobacco product use among U.S. youths.

## Methods

### National Youth Tobacco Survey Sampling Procedures

NYTS is a cross-sectional, school-based, self-administered survey of U.S. middle and high school students (*4*). The 2019 NYTS sampling frame consisted of all regular public and private schools with students enrolled in grades 6–12 in the 50 U.S. states and the District of Columbia. The sampling frame comprised data obtained from Market Data Retrieval (*5*) and the National Center for Education Statistics (*6*,*7*). Alternative schools, special education schools, U.S. Department of Defense–operated schools, Bureau of Indian Affairs schools, vocational schools, and schools with a combined total of <40 students in grades 6–12 were excluded. Participation in NYTS was voluntary at both the school and student levels; parental consent and student assent were required for NYTS participation.

The 2019 NYTS used a stratified, three-stage cluster sample design. Sampling procedures were probabilistic and conducted without replacement at all stages. Primary sampling units (PSUs), defined as individual counties, portions of a county, or groups of counties, were randomly selected within each stratum. PSUs were organized into 16 strata on the basis of urban or rural location and racial or ethnic group. Secondary sampling units, defined as schools or linked schools, were randomly selected within each PSU. The third and final sampling stage consisted of randomly selected classes. All students in the selected classes were eligible to participate in the survey; however, students who were unable to complete the questionnaire without special assistance were excluded.

### Data Collection and Processing

Since its inception in 1999, NYTS had been conducted via paper and pencil questionnaires. In 2019, NYTS for the first time was administered in schools using electronic data collection methods. Participants were provided with a tablet computer (Samsung Galaxy Tab A) to complete the survey. Data were collected offline using a programmed survey application, and a single class period of approximately 35–45 minutes was allotted to complete the survey. Survey administrators later established secure WiFi connections to synchronize all locally stored tablet data to a central repository via encrypted transmissions. Absent students and whole classes unavailable on the day of survey administration could participate in make-up surveys using a web-based version of the questionnaire programmed to mimic the tablet-based application. The 2019 NYTS was reviewed and approved by the Office of Management and Budget, the contracted data collectors’ institutional review board (IRB), and CDC’s IRB.

The 2019 questionnaire contained 104 questions covering demographic information, tobacco product use, knowledge and attitudes about tobacco products, protobacco and antitobacco media and advertising, access to tobacco products, nicotine dependence, cessation attempts, secondhand smoke and secondhand aerosol exposure, harm perceptions, exposure to tobacco product health warnings, and other tobacco-related topics. Tobacco product images and descriptions were displayed in a preamble before each tobacco product–specific section. Respondents did not answer all questions because of questionnaire skip patterns (e.g., nonusers of e-cigarettes skipped questions specific to current use of e-cigarettes) and the voluntary nature of the survey.

Survey administration occurred from February 15, 2019, to May 24, 2019. The final sample consisted of 325 schools, of which 251 participated (school response rate: 77.2%). A total of 19,018 student questionnaires were completed (17,197 tablet based and 1,821 web based) out of a sample of 22,153 students (student response rate: 85.8%). The overall response rate, defined as the product of the school-level and student-level response rates, was 66.3%. After exclusion of outliers, the average survey completion time was approximately 12.5 minutes. A weighting factor was applied to each student record to adjust for nonresponse and for varying probabilities of selection. Weights were adjusted to ensure that the weighted proportions of students in each grade matched national population proportions. Additional information on the NYTS sampling design, recruitment procedures, and data weighting is available (https://www.cdc.gov/tobacco/data_statistics/surveys/nyts/index.htm).

### Analyses

Statistical analyses were conducted using SAS-callable SUDAAN software (version 11.0.1; RTI International) to account for the complex sampling design (*8*). Weighted prevalence estimates and 95% confidence intervals were computed for all measures, and population totals were estimated from probability weights. Overall, 19,018 questionnaires were weighted to represent approximately 27.0 million students. On the basis of self-reported grade level, this included 8,837 middle school questionnaires (approximately 11.9 million students) and 10,097 high school questionnaires (approximately 15.0 million students); 84 questionnaires with missing information on grade level were excluded from school-level analyses. When applicable, estimates were determined overall and by self-reported sex (male or female), race and ethnicity (non-Hispanic white, non-Hispanic black, Hispanic, or non-Hispanic other), and school level (middle school or high school). Results with unweighted denominators <50 or a relative standard error >30% are not shown.

### Measures

#### Ever and Current Tobacco Product Use

Ever and current (past 30-day) use of seven tobacco products was assessed. These products were e-cigarettes, cigarettes, cigars (cigars, little cigars, and cigarillos), smokeless tobacco (chewing tobacco, snuff, dip, snus, and dissolvable tobacco), hookahs, pipe tobacco, and bidis (small brown cigarettes wrapped in a leaf). Ever use was defined as ever trying each respective product, and current use was defined as using each respective product on ≥1 day during the past 30 days. Any tobacco product use was defined as use of one or more of the seven tobacco products. Use of two or more tobacco product types was defined as use of two or more of the seven tobacco products. Any combustible tobacco product use was defined as use of one or more of the following: cigarettes, cigars, hookahs, pipe tobacco, and bidis. Current tobacco product use combinations also were assessed.

#### Frequency of Tobacco Product Use

The 2019 NYTS included questions to assess the frequency of tobacco product use among current users of the following tobacco products: e-cigarettes, cigarettes, cigars, smokeless tobacco (chewing tobacco, snuff, or dip), and hookahs. Respondents were asked, “During the past 30 days, on how many days did you (use e-cigarettes; smoke cigarettes; smoke cigars, cigarillos, or little cigars; use chewing tobacco, snuff, or dip; or smoke tobacco in a hookah or water pipe)?” Response options ranged between 0 and 30 days. Consistent with previous literature, response options were categorized as 1–5 days, 6–19 days, and 20–30 days for analysis (*9*). Frequent use was defined as using a product on ≥20 days of the past 30 days.

#### Flavored Tobacco Product Use

Flavored tobacco product use was determined by the response to the question, “Which of the following tobacco products that you used in the past 30 days were flavored to taste like menthol (mint), alcohol (wine or cognac), candy, fruit, chocolate, or other sweets?” Participants could select from a list of options to indicate the flavored tobacco product or products they had used. Among students who reported current use of each respective product, those who selected the flavored product were categorized as a flavored product user. Flavored (menthol) cigarette smoking, specifically, was ascertained from responses to two questions: 1) “During the past 30 days, were the cigarettes that you usually smoked menthol?” and 2) “During the past 30 days, what brand of cigarettes did you usually smoke?” Among current cigarette smokers, those reporting “yes” to the menthol question or who reported “Newport” or “Kool” as the usual cigarette brand were categorized as flavored (menthol) cigarette smokers.

#### Reasons for E-Cigarette Use

Reasons for e-cigarettes use were assessed by asking ever and current e-cigarette users, “What are the reasons why you have used electronic cigarettes or e-cigarettes?” Respondents could select one or more of 12 specified reasons. Those who indicated “I used them for some other reason” could specify their reason with a write-in response; analysis of these write-in responses (n = 642) was not included in this report. Reasons for use of other tobacco products were not assessed in the 2019 NYTS.

#### Exposure to Tobacco Product Marketing

Exposure to tobacco product marketing (advertisements or promotions) were assessed for four sources: retail stores; Internet; television, streaming sources, or movies; and newspapers or magazines. Exposure was assessed separately for e-cigarettes and cigarettes or other tobacco products. Participants were asked, “When you (are using the Internet; read newspapers or magazines; go to a convenience store, supermarket, or gas station; watch television or streaming services [such as Netflix, Hulu, or Amazon Prime], or go to the movies), how often do you see ads or promotions for (e-cigarettes; cigarettes or other tobacco products)?” Respondents were categorized as exposed if they responded “sometimes,” “most of the time,” or “always” or unexposed if they responded “never” or “rarely.” Persons who reported “I never go to a convenience stores, supermarket, or gas station,” “I do not use the Internet,” “I do not watch TV or streaming services or go to the movies,” or “I do not read newspapers or magazines” were set to missing.

#### Curiosity and Susceptibility

The 2019 NYTS included questions to assess curiosity about and susceptibility to the following tobacco products: e-cigarettes, cigarettes, cigars, hookahs, and smokeless tobacco products (chewing tobacco, snuff, or dip). For curiosity, respondents were asked, “Have you ever been curious about (using an e-cigarette; smoking a cigarette; smoking a cigar, cigarillo, or little cigar; using chewing tobacco, snuff, or dip; or smoking tobacco in a hookah or water pipe)?” To capture any level of curiosity, responses were recoded as curious (definitely yes, probably yes, or probably not) and not curious (definitely not). For each of these products, three questions assessed susceptibility: 1) “Do you think that you will try (an e-cigarette; a cigarette; a cigar, cigarillo, or little cigar; chewing tobacco, snuff, or dip; or smoking tobacco in a hookah or water pipe) soon?”; 2) “Do you think you will (use an e-cigarette; smoke a cigarette; smoke a cigar, cigarillo, or little cigar; use chewing tobacco, snuff, or dip; or smoke tobacco in a hookah or water pipe) in the next year?”; and 3) “If one of your best friends were to offer you (an e-cigarette; cigarette; cigar, cigarillo, or little cigar; chewing tobacco, snuff, or dip; or a hookah or water pipe with tobacco), would you (use; smoke; or try) it?” Consistent with previous literature, to differentiate between committed never tobacco product users from susceptible tobacco product users (*10*), susceptibility for each product was defined as a response other than “definitely not” to any of the three susceptibility questions or the curiosity question. Curiosity and susceptibility were assessed among respective never users of each respective tobacco product.

#### Harm Perceptions

The 2019 NYTS included questions to assess harm perceptions of the following tobacco products: e-cigarettes, cigarettes, smokeless tobacco (chewing, snuff, dip, or snus), and hookahs. All respondents were asked, “How much do you think people harm themselves when they (smoke cigarettes; use chewing tobacco, snuff, dip, or snus; use e-cigarettes; or smoke tobacco in a hookah or water pipe) some days but not every day?” Response options included “no harm,” “little harm,” “some harm,” and “a lot of harm.”

#### Urges to Use Tobacco Products

 Current users of any tobacco product were asked two questions: 1) “During the past 30 days, have you had a strong craving or felt like you really needed to use a tobacco product of any kind?” (with response options of yes or no) and 2) “How soon after you wake do you want to use a tobacco product?” (with response options of “I do not want to use tobacco products,” within 5 minutes, from 6 to 30 minutes, from >30 minutes to 1 hour, after >1 hour but <24 hours, and “I rarely want to use tobacco products”). Consistent with previous literature (*11*), responses to the second question were dichotomized according to whether the respondent wanted to use a tobacco product within the first 30 minutes of wakening (yes or no).

#### Quitting Behaviors

Current users of any tobacco product were asked two questions: 1) “Are you seriously thinking about quitting the use of all tobacco products?” (with response options of “yes, during the next 6 months”; “yes, during the next 12 months”; “yes, but not during the next 12 months”; and “no, I am not thinking about quitting the use of all tobacco products”) and 2) “During the past 12 months, how many times have you stopped using all tobacco products for one day or longer, because you were trying to quit tobacco products for good?” (with response options of one time, two times, three to five times, six to nine times, ≥10 times, and “I did not try to quit during the past 12 months”). Responses were dichotomized by whether the respondent was seriously thinking about quitting (yes or no) or made past-year quit attempts (more than one time or did not try quitting).

## Results

### Ever Tobacco Product Use

In 2019, 40.5% of U.S. middle and high school students (10.9 million) reported having ever tried a tobacco product (Table 1). Among ever tobacco product users, 58.5% had ever tried a combustible tobacco product and 53.8% had ever tried two or more tobacco product types. Overall, 53.3% of high school students (8.0 million) and 24.3% of middle school students (2.9 million) reported ever using any tobacco product. E-cigarettes were the most commonly ever used tobacco product among U.S middle and high school students overall (35.0%; 9.4 million), among females (35.4%) and males (35.7%), and among non-Hispanic whites (38.2%), Hispanics (35.4%), non-Hispanic blacks (27.0%), and non-Hispanics of other races (24.4%).

**TABLE 1:** Percentage of middle and high school students who reported ever using tobacco products, by product,* school level, sex, and race/ethnicity — National Youth Tobacco Survey, United States, 2019

Tobacco product	Sex		Race/Ethnicity				Total	
	Female	Male	White, non-Hispanic	Black, non-Hispanic	Hispanic^†^	Other, non-Hispanic		
	% (95% CI)	% (95% CI)	% (95% CI)	% (95% CI)	% (95% CI)	% (95% CI)	% (95% CI)	Estimated weighted no.^§^
**Overall**								
E-cigarettes	34.5 (32.1–36.9)	35.7 (32.9–38.5)	38.2 (35.7–40.8)	27.0 (24.1–30.1)	35.4 (32.6–38.3)	24.4 (20.2–29.1)	**35.0 (32.9–37.2)**	**9,430,000**
Cigarettes	14.2 (12.6–15.9)	18.3 (15.2–22.0)	18.4 (15.8–21.3)	11.8 (9.5–14.4)	15.8 (13.2–18.8)	9.6 (7.1–12.9)	**16.4 (14.2–18.7)**	**4,410,000**
Cigars	11.8 (10.4–13.3)	16.9 (14.0–20.2)	14.9 (12.8–17.2)	17.9 (15.2–21.1)	13.3 (11.0–16.1)	7.2 (5.2–9.9)	**14.4 (12.6–16.4)**	**3,880,000**
Smokeless tobacco	5.1 (4.1–6.4)	13.1 (10.5–16.2)	11.8 (9.5–14.6)	4.6 (3.4–6.3)	6.8 (5.3–8.5)	5.7 (4.1–8.0)	**9.2 (7.5–11.3)**	**2,480,000**
Hookahs	6.9 (5.8–8.2)	7.3 (5.4–9.9)	5.8 (4.5–7.3)	10.1 (7.5–13.4)	8.7 (6.8–10.9)	6.2 (4.6–8.4)	**7.1 (5.8–8.6)**	**1,910,000**
Pipe tobacco	1.8 (1.4–2.1)	3.8 (2.5–5.8)	3.2 (2.2–4.6)	—^¶^	2.5 (1.8–3.4)	—	**2.8 (2.1–3.8)**	**750,000**
Any tobacco product**	39.6 (37.1–42.3)	41.5 (38.4–44.6)	42.4 (39.5–45.4)	38.4 (34.3–42.7)	40.8 (37.8–43.9)	29.3 (25.0–33.9)	**40.5 (38.2–43.0)**	**10,930,000**
Any combustible tobacco product^††^	21.7 (19.8–23.8)	25.6 (22.3–29.1)	23.7 (21.1–26.6)	27.1 (23.2–31.4)	24.0 (21.3–27.0)	15.7 (12.5–19.7)	**23.7 (21.5–26.1)**	**6,390,000**
Two or more tobacco products^§§^	19.2 (17.4–21.1)	24.2 (21.0–27.9)	23.1 (20.4–26.0)	20.7 (17.8–24.0)	21.7 (19.1–24.6)	13.0 (10.3–16.2)	**21.8 (19.6–24.1)**	**5,870,000**
**High school**								
E-cigarettes	46.2 (43.4–49.1)	47.7 (43.8–51.7)	52.2 (49.4–54.9)	33.8 (29.8–38.0)	44.9 (41.1–48.8)	33.4 (27.5–39.9)	**46.9 (44.2–49.7)**	**7,040,000**
Cigarettes	19.2 (16.7–21.9)	25.7 (20.7–31.3)	25.9 (22.2–30.0)	14.5 (11.2–18.6)	21.1 (17.0–25.8)	12.8 (8.6–18.6)	**22.6 (19.3–26.2)**	**3,390,000**
Cigars	16.8 (14.9–18.8)	24.5 (20.1–29.4)	22.0 (18.9–25.5)	24.6 (21.5–27.8)	17.9 (14.4–22.0)	10.0 (6.9–14.2)	**20.8 (18.1–23.7)**	**3,110,000**
Smokeless tobacco	6.6 (4.9–8.8)	17.8 (14.0–22.2)	16.2 (12.9–20.1)	5.7 (3.8–8.5)	8.1 (6.1–10.7)	7.9 (5.5–11.1)	**12.5 (9.9–15.6)**	**1,870,000**
Hookahs	9.4 (7.7–11.4)	10.4 (7.7–14.8)	8.1 (6.0–10.8)	14.8 (11.3–19.2)	11.8 (9.0–15.3)	8.5 (6.1–11.8)	**9.9 (7.9–12.4)**	**1,480,000**
Pipe tobacco	2.0 (1.6–2.6)	5.5 (3.4–8.8)	4.5 (3.0–6.8)	—	2.9 (1.9–4.6)	—	**3.8 (2.6–5.6)**	**570,000**
Any tobacco product	52.4 (49.5–55.2)	54.2 (50.1–58.2)	56.9 (53.7–60.1)	47.6 (43.1–52.1)	51.0 (47.2–54.8)	38.9 (32.9–45.2)	**53.3 (50.5–56.1)**	**8,010,000**
Any combustible tobacco product	28.9 (26.2–31.7)	35.0 (30.2–40.1)	32.9 (29.2–36.8)	35.3 (31.1–39.7)	30.9 (27.0–35.2)	20.5 (15.3–26.8)	**32.1 (29.0–35.4)**	**4,820,000**
Two or more tobacco products	25.9 (23.5–28.5)	33.6 (28.7–38.8)	32.2 (28.6–36.1)	27.5 (24.0–31.3)	28.3 (24.4–32.5)	17.3 (13.0–22.7)	**29.9 (26.8–33.2)**	**4,490,000**
**Middle school**								
E-cigarettes	19.9 (18.1–21.8)	19.9 (17.9–22.1)	19.3 (17.3–21.4)	18.4 (15.5–21.8)	23.9 (21.6–26.4)	12.6 (9.4–16.8)	**19.9 (18.3–21.6)**	**2,350,000**
Cigarettes	8.0 (6.6–9.7)	8.7 (7.5–10.0)	8.2 (6.7–9.9)	8.4 (6.3–11.0)	9.2 (7.6–11.1)	—	**8.4 (7.2–9.7)**	**990,000**
Cigars	5.5 (4.3–7.2)	7.0 (5.9–8.3)	5.1 (4.2–6.3)	9.6 (6.8–13.7)	7.7 (6.3–9.3)	—	**6.3 (5.2–7.6)**	**740,000**
Smokeless tobacco	3.1 (2.3–4.3)	6.9 (5.6–8.4)	5.9 (4.5–7.7)	—	5.0 (3.9–6.5)	—	**5.0 (4.1–6.2)**	**590,000**
Hookahs	3.8 (3.0–4.8)	3.3 (2.6–4.2)	2.7 (2.0–3.6)	4.2 (2.9–5.9)	4.9 (3.7–6.5)	—	**3.5 (2.9–4.4)**	**410,000**
Pipe tobacco	1.4 (1.1–1.8)	1.7 (1.3–2.2)	1.4 (1.0–2.0)	—	—	—	**1.6 (1.3–1.9)**	**180,000**
Any tobacco product	23.8 (21.5–26.2)	24.8 (22.5–27.2)	22.7 (20.4–25.3)	26.9 (22.8–31.5)	28.3 (25.7–31.0)	16.3 (12.4–21.2)	**24.3 (22.4–26.3)**	**2,880,000**
Any combustible tobacco product	12.8 (11.1–14.8)	13.2 (11.5–15.0)	11.2 (9.7–13.0)	16.8 (12.9–21.6)	15.5 (13.4–17.8)	9.3 (6.2–13.8)	**13.0 (11.5–14.7)**	**1,540,000**
Two or more tobacco products	10.9 (9.3–12.7)	12.0 (10.5–13.7)	10.6 (8.9–12.6)	12.2 (9.6–15.2)	13.8 (11.9–15.9)	—	**11.5 (10.1–13.0)**	**1,360,000**

**Abbreviations:** CI = confidence interval; e-cigarettes = electronic cigarettes.

* Ever use of e-cigarettes was determined by asking, “Have you ever used an e-cigarette, even once or twice?” Ever use of cigarettes was determined by asking, “Have you ever tried cigarette smoking, even one or two puffs?” Ever use of cigars was determined by asking, “Have you ever tried smoking cigars, cigarillos, or little cigars, such as Swisher Sweets, Black and Mild, Garcia y Vega, Cheyenne, White Owl, or Dutch Masters, even one or two puffs?” Smokeless tobacco was defined as use of chewing tobacco, snuff, dip, snus, or dissolvable tobacco products. Ever use of smokeless tobacco was determined by asking the following question for use of chewing tobacco, snuff, and dip: “Have you ever used chewing tobacco, snuff, or dip, such as Copenhagen, Grizzly, Skoal, or Longhorn, even just a small amount?” and the following question for use of snus and dissolvable tobacco products: “Which of the following tobacco products have you ever tried, even just one time?” Responses from these questions were combined to derive overall smokeless tobacco use. Ever use of hookahs was determined by asking, “Have you ever tried smoking tobacco in a hookah or water pipe, even one or two puffs?” Ever use of pipe tobacco (not hookahs) was determined by asking, “Which of the following tobacco products have you ever tried, even just one time?” Because of missing data on the ever use questions, denominators for each tobacco product might be different.

^†^ Hispanic persons could be of any race.

^§^ Estimated weighted total number of ever tobacco product users was rounded down to the nearest 10,000 persons. Overall estimates were reported among 19,018 U.S. middle and high school students. School level was determined by self-reported grade level: high school (grades 9–12; n = 10,097) and middle school (grades 6–8; n = 8,837). Overall estimates might not directly total to sums of corresponding subgroup estimates because of rounding or inclusion of students who did not self-report sex, race/ethnicity, or grade level.

^¶^ Data were statistically unreliable because of unweighted denominator <50 or a relative standard error >30%.

** Any tobacco product use was defined as ever use of any tobacco product (e-cigarettes, cigarettes, cigars, smokeless tobacco, hookahs, pipe tobacco, or bidis [small brown cigarettes wrapped in a leaf]), even just one time.

^††^ Any combustible tobacco product use was defined as ever use of cigarettes, cigars, hookahs, pipe tobacco, or bidis.

^§§^ Defined as ever use of two or more tobacco products (e-cigarettes, cigarettes, cigars, smokeless tobacco, hookahs, pipe tobacco, or bidis).

### Current Tobacco Product Use

**FIGURE 1:**
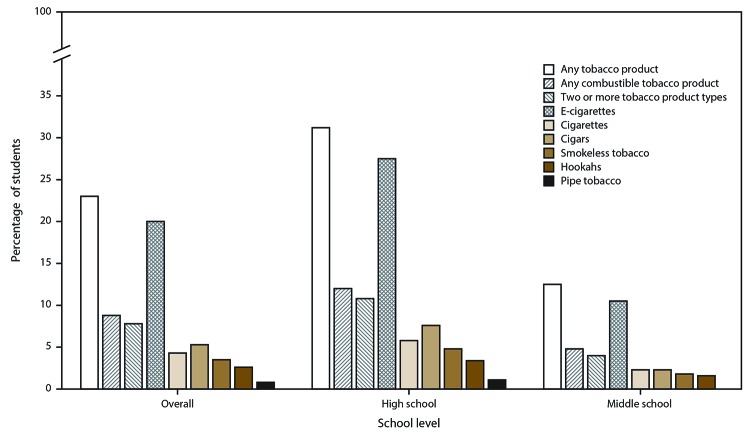
Percentage of middle and high school students who currently use any tobacco product,* any combustible tobacco product,^†^ two or more tobacco product types,^§^ and selected tobacco products, by school level^¶^ and overall — National Youth Tobacco Survey, United States, 2019 **Abbreviation:** E-cigarettes = electronic cigarettes. * Any tobacco product use was defined as use of e-cigarettes, cigarettes, cigars, hookahs, smokeless tobacco (chewing tobacco, snuff, dip, snus, or dissolvable tobacco products), pipe tobacco, or bidis (small brown cigarettes wrapped in a leaf) on ≥1 day during the past 30 days. ^†^ Any combustible tobacco product use was defined as use of cigarettes, cigars, hookahs, pipe tobacco, or bidis on ≥1 day during the past 30 days. ^§^ Defined as use of two or more tobacco products (e-cigarettes, cigarettes, cigars, hookahs, smokeless tobacco, pipe tobacco, or bidis) on ≥1 day during the past 30 days. ^¶^ On the basis of self-reported grade level among high school students (grades 9–12) and middle school students (grades 6–8), respectively. Current use of pipe tobacco among middle school students is not shown because of unweighted denominator <50 or a relative standard error >30%.

**FIGURE 2:**
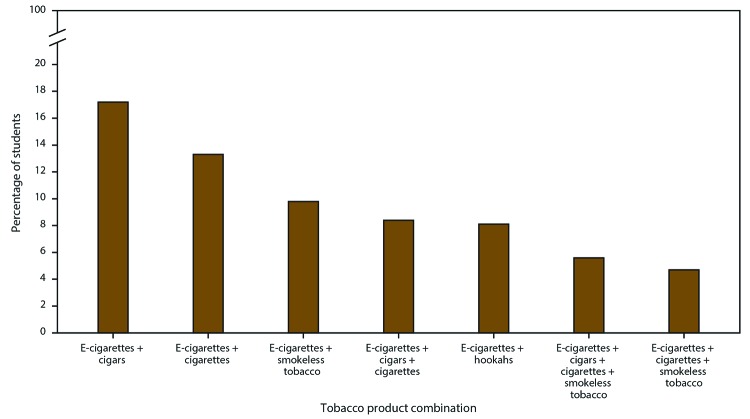
Percentage of middle and high school students who reported current use of two or more tobacco product types,* by product combination^†,§^ — National Youth Tobacco Survey, United States, 2019 **Abbreviation:** E-cigarettes = electronic cigarettes. * Percentages were calculated among youths who used two or more of the following seven tobacco product types on ≥1 day during the past 30 days: e-cigarettes, cigarettes, cigars, smokeless tobacco (chewing tobacco, snuff, dip, snus, or dissolvable tobacco products), hookahs, pipe tobacco, or bidis (small brown cigarettes wrapped in a leaf). ^†^ A total of 120 distinct combinations were assessed (21 two-product type combinations, 35 three-product type combinations, 35 four-product type combinations, 21 five-product type combinations, seven six-product type combinations, and one seven-product type combination). ^§^ All other 113 tobacco product combinations not shown were statistically unreliable because of unweighted denominator <50 or a relative standard error >30%.

Overall, 23.0% of middle and high school students (6.2 million) reported current (past 30-day) use of any tobacco product (Table 2). Among current tobacco product users, 38.3% currently used any combustible tobacco product and 33.9% currently used two or more tobacco product types. E-cigarettes were the most commonly used tobacco product overall (20.0%; 5.4 million), followed by cigars (5.3%), cigarettes (4.3%), smokeless tobacco (3.5%), hookahs (2.6%), and pipe tobacco (<1.0%) (Figure 1). Among current tobacco product users, 55.5% reported use of e-cigarettes only. Moreover, e-cigarettes were the most commonly used product in combination with other tobacco products; among students who reported current use of two or more tobacco products, 17.2% reported current use of e-cigarettes and cigars, 13.3% reported current use of cigarettes and cigarettes, and 9.8% reported current use of e-cigarettes and smokeless tobacco (Figure 2).

**TABLE 2:** Percentage of middle and high school students who reported current (past 30-day) tobacco product use, by product,* school level, sex, and race/ethnicity — National Youth Tobacco Survey, United States, 2019

Tobacco product	Sex		Race/Ethnicity				Total	
	Female	Male	White, non-Hispanic	Black, non-Hispanic	Hispanic^†^	Other, non-Hispanic		
	% (95% CI)	% (95% CI)	% (95% CI)	% (95% CI)	% (95% CI)	% (95% CI)	% (95% CI)	Estimated weighted no.^§^
**Overall**								
E-cigarettes	20.0 (18.3–21.8)	20.1 (18.5–21.9)	23.1 (21.1–25.1)	13.6 (11.5–16.1)	18.7 (16.9–20.7)	13.6 (10.9–16.9)	**20.0 (18.6–21.6)**	**5,380,000**
Cigars	4.3 (3.7–5.1)	6.3 (5.4–7.2)	5.1 (4.3–6.1)	8.6 (7.0–10.6)	4.8 (3.9–5.9)	—^¶^	**5.3 (4.6–6.1)**	**1,430,000**
Cigarettes	3.4 (2.7–4.1)	5.1 (4.0–6.4)	5.0 (3.9–6.4)	3.1 (2.3–4.1)	3.6 (2.8–4.5)	—	**4.3 (3.5–5.2)**	**1,150,000**
Smokeless tobacco	1.4 (1.0–1.9)	5.5 (4.4–6.9)	4.5 (3.4–6.0)	—	2.4 (1.9–3.0)	—	**3.5 (2.8–4.4)**	**940,000**
Hookahs	2.6 (2.1–3.2)	2.6 (2.0–3.3)	1.9 (1.4–2.5)	4.5 (3.3–6.1)	3.3 (2.5–4.4)	—	**2.6 (2.1–3.1)**	**690,000**
Pipe tobacco	—	1.1 (0.8–1.6)	0.9 (0.6–1.4)	—	—	—	**0.8 (0.6–1.1)**	**210,000**
Any tobacco product**	22.5 (20.8–24.3)	23.5 (21.6–25.4)	25.3 (23.2–27.6)	19.6 (17.0–22.4)	22.0 (20.1–24.0)	15.3 (12.3–18.9)	**23.0 (21.4–24.6)**	**6,200,000**
Any combustible tobacco product^††^	7.8 (6.9–8.8)	9.8 (8.5–11.2)	8.5 (7.2–10.0)	12.0 (10.1–14.3)	8.8 (7.7–9.9)	5.5 (3.9–7.8)	**8.8 (7.8–9.9)**	**2,380,000**
Two or more tobacco products^§§^	6.1 (5.4–6.9)	9.4 (8.2–10.9)	8.5 (7.2–10.1)	8.2 (6.6–10.0)	6.9 (6.1–7.8)	5.0 (3.7–6.7)	**7.8 (6.9–8.9)**	**2,110,000**
**High school**								
E-cigarettes	27.4 (25.0–29.9)	27.6 (25.1–30.3)	32.4 (29.8–35.2)	17.7 (14.5–21.4)	23.2 (20.6–26.0)	18.6 (14.6–23.3)	**27.5 (25.3–29.7)**	**4,110,000**
Cigars	6.2 (5.2–7.3)	9.0 (7.7–10.5)	7.6 (6.2–9.3)	12.3 (10.2–14.7)	6.2 (5.0–7.6)	—	**7.6 (6.6–8.8)**	**1,140,000**
Cigarettes	4.1 (3.1–5.4)	7.3 (5.7–9.4)	7.1 (5.4–9.2)	—	3.8 (2.9–5.0)	—	**5.8 (4.6–7.3)**	**860,000**
Smokeless tobacco	1.8 (1.2–2.7)	7.5 (5.8–9.8)	6.5 (4.8–8.8)	—	2.6 (2.0–3.5)	—	**4.8 (3.7–6.3)**	**720,000**
Hookahs	3.2 (2.5–4.1)	3.6 (2.7–4.6)	2.5 (1.8–3.3)	6.4 (4.7–8.7)	4.0 (3.0– 5.5)	—	**3.4 (2.7–4.2)**	**500,000**
Pipe tobacco	––	1.5 (1.0–2.3)	1.3 (0.8–2.0)	—	—	—	**1.1 (0.8–1.5)**	**160,000**
Any tobacco product	30.6 (28.4–33.0)	31.8 (29.1–34.6)	35.6 (32.7–38.6)	25.4 (22.2–28.9)	26.6 (24.1–29.2)	20.7 (16.4–25.7)	**31.2 (29.1–33.5)**	**4,690,000**
Any combustible tobacco product	10.2 (8.8–11.7)	13.6 (11.7–15.8)	11.9 (10.0–14.2)	16.8 (14.4–19.5)	10.3 (8.9–11.9)	7.3 (4.8–11.0)	**12.0 (10.6–13.6)**	**1,800,000**
Two or more tobacco products	8.0 (6.9–9.3)	13.4 (11.4–15.6)	12.0 (10.0–14.4)	11.5 (9.4–14.1)	8.5 (7.3–9.8)	—	**10.8 (9.4–12.4)**	**1,620,000**
**Middle school**								
E-cigarettes	10.8 (9.4–12.4)	10.2 (8.8–11.9)	10.3 (8.8–12.0)	8.6 (6.6–11.1)	13.1 (11.2–15.3)	—	**10.5 (9.4–11.8)**	**1,240,000**
Cigars	2.0 (1.4–2.8)	2.7 (2.1–3.4)	1.8 (1.2–2.5)	—	3.1 (2.2–4.3)	—	**2.3 (1.9–2.9)**	**270,000**
Cigarettes	2.5 (1.8–3.4)	2.1 (1.6–2.7)	2.1 (1.5–3.1)	—	3.1 (2.2–4.3)	—	**2.3 (1.8–2.9)**	**270,000**
Smokeless tobacco	—	2.7 (2.1–3.5)	1.9 (1.4–2.7)	—	—	—	**1.8 (1.4–2.2)**	**210,000**
Hookahs	1.8 (1.2–2.6)	1.3 (1.0–1.8)	—	—	2.4 (1.6–3.7)	—	**1.6 (1.2–2.1)**	**180,000**
Pipe tobacco	—	—	—	—	—	—	**—**	**—**
Any tobacco product	12.4 (10.8–14.1)	12.5 (10.9–14.3)	11.4 (9.8–13.2)	12.3 (10.0–15.0)	16.1 (14.1–18.4)	—	**12.5 (11.2–13.9)**	**1,470,000**
Any combustible tobacco product	4.9 (3.8–6.2)	4.6 (3.9–5.5)	3.8 (3.0–4.9)	6.1 (4.5–8.1)	6.6 (5.4–8.1)	—	**4.8 (4.0–5.7)**	**560,000**
Two or more tobacco products	3.7 (2.9–4.7)	4.2 (3.5–5.1)	3.8 (2.9–5.0)	3.9 (3.0–5.1)	5.0 (4.0–6.2)	—	**4.0 (3.3–4.7)**	**470,000**

**Abbreviations:** CI = confidence interval; e-cigarettes = electronic cigarettes.

* Past 30-day use of e-cigarettes was determined by asking, “During the past 30 days, on how many days did you use e-cigarettes?” Past 30-day use of cigarettes was determined by asking, “During the past 30 days, on how many days did you smoke cigarettes?” Past 30-day use of cigars was determined by asking, “During the past 30 days, on how many days did you smoke cigars, cigarillos, or little cigars?” Smokeless tobacco was defined as use of chewing tobacco, snuff, dip, snus, or dissolvable tobacco products. Past 30-day use of smokeless tobacco was determined by asking the following question for use of chewing tobacco, snuff, and dip: “During the past 30 days, on how many days did you use chewing tobacco, snuff, or dip?” and the following question for use of snus and dissolvable tobacco products: “In the past 30 days, which of the following products did you use on at least one day?” Responses from these questions were combined to derive overall smokeless tobacco use. Past 30-day use of hookahs was determined by asking, “During the past 30 days, on how many days did you smoke tobacco in a hookah or water pipe?” Past 30-day use of pipe tobacco (not hookahs) was determined by asking, “In the past 30 days, which of the following products have you used on at least one day?” Because of missing data on the past 30-day use questions, denominators for each tobacco product might be different.

^†^ Hispanic persons could be of any race.

^§^ Estimated weighted total number of current tobacco product users was rounded down to the nearest 10,000 persons. Overall estimates were reported among 19,018 U.S. middle and high school students. School level was determined by self-reported grade level: high school (grades 9–12; n = 10,097) and middle school (grades 6–8; n = 8,837). Overall estimates might not directly total to sums of corresponding subgroup estimates because of rounding or inclusion of students who did not self-report sex, race/ethnicity, or grade level.

^¶^ Data were statistically unreliable because of unweighted denominator <50 or a relative standard error >30%.

** Any tobacco product use was defined as use of any tobacco product (e-cigarettes, cigarettes, cigars, smokeless tobacco, hookahs, pipe tobacco, or bidis [small brown cigarettes wrapped in a leaf]) on ≥1 day during the past 30 days.

^††^ Any combustible tobacco product use was defined as use of cigarettes, cigars, hookahs, pipe tobacco, or bidis on ≥1 day during the past 30 days.

^§§^ Defined as use of two or more tobacco products (e-cigarettes, cigarettes, cigars, smokeless tobacco, hookahs, pipe tobacco, or bidis) on ≥1 day during the past 30 days.

Among high school students, 31.2% (4.7 million) reported current use of any tobacco product. Among these current tobacco product users, 38.5% currently used any combustible tobacco product and 34.6% reported current use of two or more tobacco product types. Among high school students, e-cigarettes were the most commonly used tobacco product (27.5%), followed by cigars (7.6%), cigarettes (5.8%), smokeless tobacco (4.8%), hookahs (3.4%), and pipe tobacco (1.1%).

Among middle school students, 12.5% (1.5 million) reported current use of any tobacco product. Among these current tobacco product users, 38.4% used any combustible tobacco product and 32.0% reported current use of two or more tobacco product types. Among middle school students, e-cigarettes were the most commonly used tobacco product (10.5%), followed by cigarettes and cigars (both 2.3%), smokeless tobacco (1.8%), and hookahs (1.6%).

### Frequency of Tobacco Product Use

Among middle and high school students who were current users of each product, the prevalence of frequent tobacco product use (≥20 days of the past 30 days) was 34.1% of smokeless tobacco users (270,000), 30.4% of e-cigarette users (1.6 million), 28.9% of cigarette smokers (330,000), 18.6% of hookah smokers (120,000), and 16.8% of cigar smokers (240,000) (Table 3). Most current tobacco product users reported using tobacco products on 1–5 days of the past 30 days, including 69.1% of hookah smokers, 68.7% of cigar smokers, 55.9% of cigarette smokers, 50.8% of e-cigarette users, and 49.3% of smokeless tobacco users.

**TABLE 3:** Frequency of use* among middle and high school students currently using cigarettes, e-cigarettes, cigars, smokeless tobacco, and hookahs — National Youth Tobacco Survey, United States, 2019

Days of use	E-cigarettes		Cigarettes		Cigars		Smokeless tobacco		Hookahs	
	% (95% CI)^†^	Estimated no.^§^	% (95% CI)	Estimated no.	% (95% CI)	Estimated no.	% (95% CI)	Estimated no.	% (95% CI)	Estimated weighted no.
**Overall** ^¶^										
1–5	50.8 (48.1–53.4)	2,730,000	55.9 (49.6–62.1)	640,000	68.7 (63.9–73.1)	980,000	49.3 (43.3–55.4)	390,000	69.1 (62.8–74.7)	480,000
6–19	18.8 (17.5–20.3)	1,010,000	15.2 (12.3–18.7)	170,000	14.5 (12.0–17.5)	200,000	16.6 (12.7–21.4)	130,000	12.4 (8.8–17.3)	80,000
20–30	30.4 (27.7–33.3)	1,630,000	28.9 (23.1–35.5)	330,000	16.8 (12.7–21.9)	240,000	34.1 (28.2–40.5)	270,000	18.6 (13.1–25.6)	120,000
High school**										
1–5	46.4 (43.6–49.3)	1,910,000	51.5 (44.2–58.7)	440,000	68.6 (63.5–73.3)	780,000	44.0 (37.1–51.2)	270,000	69.2 (61.7–75.8)	350,000
6–19	19.4 (17.8–21.1)	790,000	16.0 (12.3–20.6)	130,000	14.1 (11.4–17.4)	160,000	18.0 (13.7–23.4)	110,000	13.2 (8.6–19.6)	60,000
20–30	34.2 (31.2–37.3)	1,400,000	32.5 (25.3–40.5)	280,000	17.3 (13.0–22.7)	190,000	37.9 (30.3–46.2)	230,000	17.6 (12.0–25.1)	80,000
**Middle school** ^††^										
1–5	65.3 (61.0–69.5)	810,000	70.1 (62.2–76.9)	190,000	69.0 (58.3–78.0)	190,000	66.0 (54.5–75.9)	110,000	68.1 (58.8–76.1)	120,000
6–19	16.7 (13.5–20.4)	200,000	—^§§^	—	—	—	—	—	—	—
20–30	18.0 (15.2–21.2)	220,000	—	—	—	—	—	—	—	—

**Abbreviations:** CI = confidence interval; e-cigarettes = electronic cigarettes.

* Frequency of current use of e-cigarettes, cigarettes, cigars (defined as cigars, cigarillos, or little cigars), smokeless tobacco (defined as chewing tobacco, snuff, or dip; frequency of use was not assessed for snus or dissolvable tobacco products), and hookahs was determined by asking participants on how many days they smoked or used each of these tobacco products during the past 30 days. Respondents could enter a valid response of 0–30 days.

^†^ Reported among respective current (past 30-day) users for each product. Past 30-day use of e-cigarettes was determined by asking, “During the past 30 days, on how many days did you use e-cigarettes?” Past 30-day use of cigarettes was determined by asking, “During the past 30 days, on how many days did you smoke cigarettes?” Past 30-day use of cigars was determined by asking, “During the past 30 days, on how many days did you smoke cigars, cigarillos, or little cigars?” Past 30-day use of smokeless tobacco was determined by asking the following question for use of chewing tobacco, snuff, and dip: “During the past 30 days, on how many days did you use chewing tobacco, snuff, or dip?” Past 30-day use of hookahs was determined by asking, “During the past 30 days, on how many days did you smoke tobacco in a hookah or water pipe?”

^§^ Estimated weighted total number of users was rounded down to the nearest 10,000 persons. Overall estimates might not directly total to sums of corresponding subgroup estimates because of rounding or inclusion of students who did not self-report grade level.

^¶^ Calculated among total current users of e-cigarettes (n = 3,628), cigarettes (n = 748), cigars (n = 930), smokeless tobacco (n = 531), and hookahs (n = 477).

** Calculated among current high school tobacco product users (self-reported grades 9–12) of e-cigarettes (n = 2,709), cigarettes (n = 549), cigars (n = 727), smokeless tobacco (n = 399), and hookahs (n = 334).

^††^ Calculated among current middle school tobacco product users (self-reported grades 6–8) of e-cigarettes (n = 902), cigarettes (n = 190), cigars (n =197), smokeless tobacco (n = 125), and hookahs (n = 138).

^§§^ Data were statistically unreliable because of unweighted denominator <50 or a relative standard error >30%.

### Flavored Tobacco Product Use

In 2019, 69.6% (4.3 million) of middle and high school students who currently used tobacco products reported using at least one flavored tobacco product. E-cigarettes were the most commonly used flavored tobacco product (68.8% of current e-cigarette users; 3.7 million). The proportion of other current tobacco product users who reported flavored product use was 48.0% for smokeless tobacco, 46.7% for cigarettes (menthol only), 41.9% for cigars, 31.4% for pipe tobacco, and 31.2% for hookahs (Table 4). Among current tobacco product users, flavored tobacco product use was 72.8% among high school students and 59.6% among middle school students. Flavored tobacco product use was highest among non-Hispanic whites (76.8%) compared with students of other non-Hispanic races (68.1%), Hispanics (63.1%), and non-Hispanic blacks (48.0%). The proportion of current tobacco users who used flavored products was 68.6% among females and 70.7% among males.

**TABLE 4:** Flavored tobacco product* use among all middle and high school students and among those who reported current use† of specified tobacco products, by school level, sex, and race/ethnicity — National Youth Tobacco Survey, United States, 2019

Characteristic	Tobacco product						
	Any tobacco product^§^	E-cigarettes	Cigarettes^¶^	Cigars	Smokeless tobacco**	Hookahs	Pipe tobacco
	% (95% CI)	% (95% CI)	% (95% CI)	% (95% CI)	% (95% CI)	% (95% CI)	% (95% CI)
**Overall**							
Flavored tobacco product use among all students^††^	16.0 (14.6–17.4)	13.8 (12.5–15.1)	2.0 (1.6–2.5)	2.2 (1.9–2.6)	1.7 (1.3–2.2)	0.8 (0.6–1.1)	0.3 (0.2–0.4)
**Current tobacco product users**							
Estimated weighted no. of flavored tobacco product users^§§^	4,310,000	3,700,000	530,000	600,000	450,000	210,000	60,000
Flavored tobacco product use among current tobacco product users^¶¶^	69.6 (67.0–72.0)	68.8 (66.2–71.4)	46.7 (42.5–51.0)	41.9 (38.0–46.0)	48.0 (42.8–53.2)	31.2 (25.7–37.3)	31.4 (23.1–41.1)
**School level**							
Middle school	59.6 (56.4–62.8)	59.9 (56.0–63.7)	37.2 (29.2–45.8)	36.1 (28.2–44.9)	42.3 (33.8–51.2)	27.5 (19.2–37.7)	—***
High school	72.8 (69.7–75.6)	71.7 (68.6–74.5)	49.8 (44.8–54.8)	43.2 (39.1–47.4)	49.8 (43.5–56.2)	32.9 (26.3–40.1)	28.0 (19.9–37.7)
**Sex**							
Female	68.6 (64.9–72.1)	68.3 (64.5–71.9)	49.7 (43.1–56.2)	38.6 (32.9–44.5)	36.4 (26.9–47.0)	34.7 (26.3–44.2)	—
Male	70.7 (68.1–73.1)	69.6 (66.9–72.3)	45.1 (38.9–51.5)	44.2 (39.3–49.1)	50.9 (44.6–57.2)	27.2 (19.4–36.7)	36.2 (27.3–46.1)
**Race/Ethnicity**							
White, non-Hispanic	76.8 (74.6–78.9)	75.2 (72.6–77.6)	45.8 (40.4–51.4)	44.2 (37.5–51.2)	55.8 (49.7–61.7)	32.1 (23.0–42.9)	31.0 (18.9–46.3)
Black, non-Hispanic	48.0 (41.9–54.1)	43.1 (35.9–50.7)	39.6 (25.6–55.6)	41.2 (33.7–49.2)	—	24.5 (14.0–39.4)	—
Hispanic^†††^	63.1 (59.0–67.1)	63.0 (58.5–67.2)	50.8 (42.7–58.8)	36.5 (30.2–43.3)	29.4 (21.1–39.5)	35.6 (25.3–47.5)	46.5 (30.5–63.2)
Other, non-Hispanic	68.1 (61.6–74.0)	68.7 (61.3–75.2)	47.4 (34.8–60.2)	43.7 (33.8–54.1)	40.1 (27.2–54.5)	27.4 (15.8–43.2)	—

**Abbreviations:** CI = confidence interval; e-cigarettes = electronic cigarettes.

* Flavored tobacco product use was determined by the response to the question, “Which of the following tobacco products that you used in the past 30 days were flavored to taste like menthol (mint), alcohol (wine, cognac), candy, fruit, chocolate, or other sweets?" Participants could select from a list of options to designate the flavored tobacco products they had used. Among those who reported any use of each respective product during the past 30 days, those who selected the flavored product were categorized as flavored product users, those who did not select the flavored product were categorized as only nonflavored product users, and those who did not provide any response to the flavored product use question were assigned as missing flavor status.

^†^ Current (past 30-day) use of e-cigarettes was determined by asking, “During the past 30 days, on how many days did you use e-cigarettes?” Current use of cigarettes was determined by asking, “During the past 30 days, on how many days did you smoke cigarettes?” Current use of cigars was determined by asking, “During the past 30 days, on how many days did you smoke cigars, cigarillos, or little cigars?” Smokeless tobacco was defined as use of chewing tobacco, snuff, dip, snus, or dissolvable tobacco products. Current use of smokeless tobacco was determined by asking the following question for use of chewing tobacco, snuff, and dip: “During the past 30 days, on how many days did you use chewing tobacco, snuff, or dip?” and the following question for use of snus and dissolvable tobacco products: “In the past 30 days, which of the following products did you use on at least one day?” Responses from these questions were combined to derive overall smokeless tobacco use. Current use of hookahs was determined by asking, “During the past 30 days, on how many days did you smoke tobacco in a hookah or water pipe?” Current use of pipe tobacco (not hookahs) was determined by asking, “In the past 30 days, which of the following products have you used on at least one day?”

^§^ Any current tobacco product use was defined as use of any tobacco product (e-cigarettes, cigarettes, cigars, smokeless tobacco, hookahs, pipe tobacco, or bidis [small brown cigarettes wrapped in a leaf]) on ≥1 day during the past 30 days.

^¶^ Flavored cigarette use referred to menthol cigarettes. Menthol cigarette status was determined by asking, “Menthol cigarettes are cigarettes that taste like mint. During the past 30 days, were the cigarettes that you usually smoked menthol?” and “During the past 30 days, what brand or cigarettes did you usually smoke?” Among past 30-day cigarette smokers, those responding “yes” to the menthol question or who reported “Newport” or “Kool” as the usual cigarette brand were categorized as menthol cigarette smokers; subsequently, those who reported “no” to the menthol question and who did not report “Newport” or “Kool” brands were categorized as nonmenthol cigarette smokers; all other past 30-day cigarette smokers were assigned as missing menthol cigarette smoking status.

** Any smokeless tobacco was current use of smokeless tobacco (chewing tobacco, snuff, dip, snus, or dissolvable tobacco products) on ≥1 day during the past 30 days.

^††^ Calculated among all respondents regardless of tobacco product use status. Because of missing data, denominators for each tobacco product might be different: e-cigarettes (n = 18,914), cigarettes (n = 18,975), cigars (n = 18,958), smokeless tobacco (n = 19,018), hookahs (n = 18,948), and pipe tobacco (n = 18,821).

^§§^ Estimated weighted total number of flavored tobacco product users was rounded down to the nearest 10,000 persons.

^¶¶^ Calculated among current users of any tobacco product (n = 4,198), e-cigarettes (n = 3,628), cigarettes (n = 748), cigars (n = 930), smokeless tobacco (n = 630), hookahs (n = 477), and pipe tobacco (n = 138).

*** Data were statistically unreliable because of unweighted denominator <50 or a relative standard error >30%.

^†††^ Hispanic persons could be of any race.

### Reasons for E-Cigarette Use

Among middle and high school students who ever tried using e-cigarettes, the most common reasons for e-cigarette use were “I was curious about them” (55.3%), “friend or family member used them” (30.8%), “they are available in flavors, such as mint, candy, fruit or chocolate” (22.4%), and “I can use them to do tricks” (21.2%) (Table 5). “I was curious about them” was the most commonly reported reason among current exclusive e-cigarette users (56.1%) and students who currently used both e-cigarettes and at least one other tobacco product (38.4%) (Table 6).

**TABLE 5:** Reasons for e-cigarette use* among middle and high school students who reported ever using e-cigarettes,^†^ by school level, sex, and race/ethnicity — National Youth Tobacco Survey, United States, 2019

Reason	Overall		School level		Sex		Race/Ethnicity			
			Middle school	High school	Male	Female	White, non-Hispanic	Black, non-Hispanic	Hispanic^¶^	Other, non-Hispanic
	% (95% CI)	Estimated no.^§^	% (95% CI)	% (95% CI)	% (95% CI)	% (95% CI)	% (95% CI)	% (95% CI)	% (95% CI)	% (95% CI)
I was curious about them	55.3 (53.3–57.3)	5,110,000	57.1 (54.5–59.8)	54.8 (52.5–57.0)	52.1 (49.3–54.9)	58.9 (56.8–61.0)	53.1 (50.5–55.6)	53.7 (48.8–58.5)	61.5 (58.6–64.4)	56.4 (48.7–64.0)
Friend or family member used them	30.8 (29.1–32.6)	2,850,000	36.8 (33.7–40.0)	28.9 (26.8–31.0)	27.1 (24.6–29.7)	34.9 (32.9–37.0)	31.8 (29.6–34.1)	30.8 (26.3–35.7)	28.1 (25.6–30.7)	29.1 (21.3–38.3)
They are available in flavors, such as mint, candy, fruit, or chocolate	22.4 (20.8–24.1)	2,070,000	22.8 (20.5–25.2)	22.3 (20.4–24.3)	20.7 (17.9–23.8)	24.3 (22.4–26.3)	22.8 (21.0–24.8)	21.7 (18.4–25.4)	22.4 (19.7–25.3)	19.1 (14.0–25.5)
I can use them to do tricks	21.2 (19.5–23.0)	1,960,000	22.6 (20.7–24.6)	20.8 (18.5–23.1)	23.3 (20.3–26.5)	19.0 (17.2–20.8)	21.6 (19.8–23.6)	18.9 (15.3–23.2)	21.1 (18.3–24.2)	22.1 (15.7–30.2)
They are less harmful than other forms of tobacco, such as cigarettes	15.7 (14.3–17.2)	1,450,000	15.8 (14.0–17.7)	15.6 (13.9–17.5)	17.5 (15.4–19.7)	13.6 (12.0–15.4)	16.4 (14.7–18.2)	13.0 (9.6–17.2)	15.6 (13.3–18.1)	—**
I can use them unnoticed at home or at school	13.9 (11.4–16.8)	1,280,000	10.5 (8.6–12.7)	14.9 (11.8–18.6)	14.6 (10.3–20.2)	13.1 (11.7–14.6)	14.4 (12.1–17.0)	8.0 (5.3–11.9)	14.8 (11.0–19.6)	—
I was peer pressured into using them	10.7 (9.5–12.1)	990,000	11.1 (8.9–13.7)	10.6 (9.2–12.2)	10.7 (9.0–12.7)	10.8 (9.2–12.6)	11.8 (10.1–13.7)	8.7 (6.5–11.6)	8.8 (6.9–11.0)	—
To try to quit using other tobacco products, such as cigarettes	5.5 (4.5–6.7)	500,000	—	6.4 (5.1–7.9)	7.0 (5.7–8.5)	3.7 (2.7–5.2)	6.6 (5.2–8.4)	—	3.8 (2.9–5.0)	—
They are easier to get than other tobacco products, such as cigarettes	5.4 (4.1–7.0)	500,000	5.0 (3.8–6.5)	5.5 (4.0–7.5)	6.5 (4.5–9.4)	4.1 (3.3–5.1)	5.8 (4.2–8.0)	—	4.6 (3.7–5.6)	—
I’ve seen people on TV, online, or in movies use them	4.4 (3.8–5.0)	400,000	6.3 (5.1–7.9)	3.7 (3.2–4.4)	4.2 (3.6–5.0)	4.4 (3.6–5.4)	3.9 (3.2–4.7)	—	4.9 (3.7–6.5)	—
They cost less than other tobacco products, such as cigarettes	3.8 (3.1–4.8)	350,000	—	4.2 (3.3–5.3)	5.2 (4.0–6.7)	2.3 (1.7–3.2)	4.6 (3.5–6.1)	—	—	—
I used them for some other reason^††^	14.4 (12.4–16.6)	1,330,000	15.4 (13.2–18.0)	14.1 (11.8–16.7)	15.8 (12.8–19.3)	12.8 (11.4–14.3)	15.1 (12.6–18.0)	13.8 (10.8–17.4)	12.9 (10.8–15.4)	—

**Abbreviations:** CI = confidence interval; e-cigarettes = electronic cigarettes; TV = television.

* Assessed by the question, “What are the reasons why you have used electronic cigarettes or e-cigarettes? (Check all that apply.)” Responses were not mutually exclusive.

^†^ Assessed by the question, “Have you ever used an e-cigarette, even once or twice?” Ever users reported e-cigarette use on ≥1 day during the past 30 days (n = 6,409).

^§^ Estimated weighted total number of users was rounded down to the nearest 10,000 persons.

^¶^ Hispanic persons could be of any race.

** Data were statistically unreliable because of unweighted denominator <50 or a relative standard error >30%.

^††^ Respondents could subsequently specify a reason through a write-in option (n = 642).

**TABLE 6:** Reasons for e-cigarette use* among middle and high school students who reported using e-cigarettes and other tobacco products during the past 30 days — National Youth Tobacco Survey, United States, 2019

Reason	Use e-cigarettes only^†^		Use e-cigarettes and other tobacco products^§^	
	% (95% CI)	Estimated no.^¶^	% (95% CI)	Estimated no.
I was curious about them	56.1 (53.4–58.7)	1,900,000	38.4 (35.1–41.7)	730,000
Friend or family member used them	23.9 (21.7–26.3)	810,000	22.2 (19.6–25.1)	420,000
They are available in flavors, such as mint, candy, fruit, or chocolate	22.3 (20.3–24.5)	760,000	26.6 (23.8–29.6)	500,000
I can use them to do tricks	22.0 (20.0–24.2)	740,000	29.0 (25.6–32.7)	550,000
They are less harmful than other forms of tobacco, such as cigarettes	17.2 (15.3–19.3)	580,000	19.1 (16.7–21.9)	360,000
I can use them unnoticed at home or at school	14.5 (12.9–16.3)	490,000	22.9 (19.4–26.8)	430,000
I was peer pressured into using them	8.9 (7.7–10.3)	300,000	7.5 (5.8–9.8)	140,000
They are easier to get than other tobacco products, such as cigarettes	3.9 (3.0–5.0)	130,000	9.7 (7.9–11.8)	180,000
I’ve seen people on TV, online, or in movies use them	3.8 (3.1–4.6)	120,000	5.4 (3.9–7.4)	100,000
To try to quit using other tobacco products, such as cigarettes	2.8 (1.8–4.2)	90,000	17.0 (14.0–20.5)	320,000
They cost less than other tobacco products, such as cigarettes	2.5 (1.9–3.3)	80,000	11.6 (9.4–14.3)	220,000
I used them for some other reason**	15.9 (14.0–18.0)	540,000	22.2 (17.9–27.3)	420,000

**Abbreviations:** CI = confidence interval; e-cigarettes = electronic cigarettes; TV = television.

* Assessed by the question, “What are the reasons why you have used electronic cigarettes or e-cigarettes? (Check all that apply.)” Responses were not mutually exclusive.

^†^ Reported use of only e-cigarettes on ≥1 day during the past 30 days (n = 2,361).

^§^ Reported use of e-cigarettes and at least one other tobacco product (e-cigarettes and cigarettes, cigars, smokeless tobacco, hookahs, pipe tobacco, or bidis [small brown cigarettes wrapped in a leaf]) on ≥1 day during the past 30 days (n = 1,267).

^¶^ Estimated weighted total number of users was rounded down to the nearest 10,000 persons.

** Respondents could subsequently specify a reason through a write-in option (n = 465).

### Exposure to Tobacco Product Marketing

In 2019, NYTS data indicated that 86.3% of middle and high school students who reported contact with a potential source of tobacco product advertisements or promotions (going to a convenience store, supermarket, or gas station; using the Internet; watching television or streaming services or going to the movies; reading newspapers or magazines) reported exposure to any tobacco product marketing (Table 7). The prevalence of exposure was 79.4% among students who reported going to retail stores, 59.6% among those who reported using the Internet, 53.5% among those who reported reading newspapers or magazines, and 36.9% among those who reported watching television or streaming services or going to the movies. Overall, 69.3% of middle and high school students reported exposure to e-cigarette marketing specifically, whereas 81.7% reported exposure to cigarette or other tobacco product marketing.

**TABLE 7:** Percentage of middle and high school students who reported exposure* to sources of tobacco product marketing (advertisements or promotions), by school level, sex, and race/ethnicity — National Youth Tobacco Survey, United States, 2019

Characteristic	Retail stores^†^	Internet^§^	TV, streaming services, or movies^¶^	Newspapers or magazines**	Any source^††^
	% (95% CI)	% (95% CI)	% (95% CI)	% (95% CI)	% (95% CI)
**Exposure to any tobacco product marketing**					
**Overall**	**79.4 (78.1–80.7)**	**59.6 (58.3–60.9)**	**36.9 (35.0–38.8)**	**53.5 (51.9–55.1)**	**86.3 (85.4–87.1)**
**Estimated no.^§§^**	**20,410,000**	**15,400,000**	**9,260,000**	**7,490,000**	**22,930,000**
Sex					
Male	77.7 (75.9–79.3)	56.3 (54.2–58.5)	34.2 (31.7–36.8)	53.0 (50.5–55.5)	84.4 (83.0–85.7)
Female	81.2 (79.7–82.7)	63.1 (61.6–64.5)	39.6 (37.7–41.6)	53.9 (52.1–55.8)	88.3 (87.3–89.3)
Race/Ethnicity					
White, non-Hispanic	83.1 (81.8–84.3)	59.9 (58.3–61.5)	34.7 (32.4–37.2)	52.9 (50.6–55.1)	88.3 (87.3–89.3)
Black, non-Hispanic	75.4 (73.1–77.6)	61.4 (58.5–64.3)	46.9 (43.4–50.6)	58.3 (54.3–62.2)	86.1 (84.2–87.8)
Hispanic**^¶¶^**	76.2 (74.3–78.1)	59.6 (57.7–61.4)	38.0 (36.0–40.0)	53.8 (51.7–55.8)	84.3 (82.9–85.7)
Other, non-Hispanic	68.0 (65.0–70.9)	55.7 (52.1–59.3)	28.8 (25.7–32.1)	47.9 (43.7–52.1)	78.7 (76.0–81.3)
School level					
Middle school	77.3 (75.4–79.1)	58.2 (56.6–59.7)	35.0 (32.8–37.3)	52.9 (50.7–55.2)	85.2 (83.9–86.4)
High school	81.2 (79.7–82.6)	60.8 (59.2–62.4)	38.4 (36.1–40.7)	53.9 (51.8–56.0)	87.3 (86.2–88.3)
Exposure to e-cigarette marketing***					
**Overall**	**58.4 (56.5–60.2)**	**44.6 (43.4–45.8)**	**26.2 (24.9–27.5)**	**34.8 (33.5–36.1)**	**69.3 (67.8–70.8)**
**Estimated no.**	**15,030,000**	**11,510,000**	**6,620,000**	**5,070,000**	**18,260,000**
Sex					
Male	56.6 (54.4–58.7)	41.2 (39.5–43.0)	23.9 (22.2–25.7)	33.3 (31.2–35.5)	67.3 (65.4–69.2)
Female	60.3 (58.1–62.4)	48.1 (46.4–49.8)	28.5 (27.0–30.2)	36.4 (34.7–38.0)	71.5 (69.6–73.2)
Race/Ethnicity					
White, non-Hispanic	62.9 (60.7–65.0)	46.2 (44.6–47.9)	26.0 (24.2–27.8)	34.8 (33.0–36.7)	72.6 (70.8–74.2)
Black, non-Hispanic	52.2 (49.5–55.0)	42.6 (40.0–45.2)	30.0 (27.4–32.7)	36.0 (32.7–39.4)	66.5 (64.3–68.6)
Hispanic	54.0 (51.9–56.2)	43.4 (41.8–45.1)	26.3 (24.6–28.1)	35.5 (33.7–37.3)	66.2 (64.1–68.2)
Other, non-Hispanic	48.8 (44.7–52.9)	41.8 (38.7–45.1)	19.4 (17.1–21.9)	31.9 (28.3–35.8)	62.4 (58.9.–65.7)
School level					
Middle school	53.8 (51.4–56.2)	41.5 (40.0–43.1)	24.3 (22.6–26.0)	33.4 (31.4–35.4)	65.7 (63.8–67.5)
High school	62.1 (60.0–64.2)	47.1 (45.6–48.6)	27.7 (26.0–29.4)	35.8 (34.0–37.7)	72.3 (70.6–74.0)
**Exposure to cigarette or other tobacco product marketing^†††^**					
**Overall**	**72.8 (71.3–74.3)**	**43.1 (41.4–44.7)**	**26.8 (25.0–28.6)**	**36.7 (34.9–38.6)**	**81.7 (80.7–82.7)**
**Estimated no.**	**18,670,000**	**11,180,000**	**6,770,000**	**5,410,000**	**21,630,000**
Sex					
Male	70.8 (68.8–72.8)	40.6 (38.2–43.1)	24.8 (22.6–27.1)	36.9 (34.1–39.8)	79.5 (78.0–81.0)
Female	75.0 (73.4–76.5)	45.6 (44.0–47.3)	28.8 (27.0–30.6)	36.4 (34.6–38.3)	84.1 (82.9–85.2)
Race/Ethnicity					
White, non-Hispanic	77.1 (75.6–78.5)	41.7 (39.6–43.9)	24.3 (22.2–26.5)	36.0 (33.7–38.4)	83.7 (82.5–84.9)
Black, non-Hispanic	67.9 (65.2–70.4)	48.7 (46.2–51.2)	37.0 (34.0–40.2)	41.7 (37.1–46.4)	82.1 (80.1–83.9)
Hispanic	69.2 (66.8–71.5)	44.2 (42.4–46.0)	27.7 (25.8–29.7)	36.9 (35.1–38.7)	79.7 (78.1–81.3)
Other, non-Hispanic	61.1 (57.7–64.5)	38.8 (35.3–42.3)	21.2 (18.6–24.1)	30.3 (26.7–34.2)	72.9 (70.2–75.5)
School level					
Middle school	71.7 (69.7–73.5)	44.5 (42.8–46.2)	25.9 (24.0–28.0)	36.8 (34.8–38.8)	81.5 (80.1–82.8)
High school	73.9 (72.1–75.6)	42.0 (39.8–44.2)	27.5 (25.4–29.7)	36.7 (34.4–39.1)	82.0 (80.7–83.3)

**Abbreviations:** CI = confidence interval; e-cigarettes = electronic cigarettes; TV = television.

* Exposure to tobacco product marketing was assessed for each of four marketing sources (retail stores; Internet; television, streaming sources, or movies; and newspapers or magazines) and any source combined. For each source, exposure was assessed separately for 1) e-cigarettes and 2) cigarettes or other tobacco products. A composite measure of exposure to any tobacco product marketing was also assessed.

^†^ Assessed by the questions, “When you go to a convenience store, supermarket, or gas station, how often do you see ads or promotions for (e-cigarettes; cigarettes or other tobacco products)?” Response options for both questions included, “I never go to a convenience store, supermarket, or gas station,” “never,” “rarely,” “sometimes,” “most of the time,” and “always.” Respondents were categorized as exposed if they reported “sometimes,” “most of the time,” or “always”; respondents were categorized as unexposed if they reported “never” or “rarely.” Persons who reported “I never go to a convenience store, supermarket, or gas station” were set to missing and excluded from product-specific analyses. Total unweighted denominators included n = 18,110 (e-cigarettes) and n = 18,034 (cigarettes or other tobacco products). A composite measure of exposure to any tobacco product marketing when going to retail stores (overall) was assessed among respondents categorized as exposed for at least one measure or unexposed across both measures (n = 18,072).

^§^ Assessed by the questions, “When you are using the Internet, how often do you see ads or promotions for (e-cigarettes; cigarettes or other tobacco products)?” Response options for both questions included, “I do not use the Internet,” “never,” “rarely,” “sometimes,” “most of the time,” and “always.” Respondents were categorized as exposed if they reported “sometimes,” “most of the time,” or “always”; respondents were categorized as unexposed if they reported “never” or “rarely.” Persons who reported “I do not use the Internet” were set to missing and excluded from product-specific analyses. Total unweighted denominators included n = 18,175 (e-cigarettes) and n = 18,286 (cigarettes or other tobacco products). A composite measure of exposure to any tobacco product marketing when using the Internet (overall) was assessed among respondents categorized as exposed to marketing for at least one measure or unexposed across both measures (n = 18,173).

^¶^ Assessed by the questions, “When you watch TV or streaming services (such as Netflix, Hulu, or Amazon Prime), or go to the movies, how often do you see ads or promotions for (e-cigarettes; cigarettes or other tobacco products)?” Response options for both questions included, “I do not watch TV or streaming services, or go to the movies,” “never,” “rarely,” “sometimes,” “most of the time,” and “always.” Respondents were categorized as exposed if they reported “sometimes,” “most of the time,” or “always”; respondents were categorized as unexposed if they reported “never” or “rarely.” Persons who reported “I do not watch TV or streaming services, or go to the movies” were set to missing and excluded from product-specific analyses. Total unweighted denominators for exposure to tobacco product marketing included n = 17,798 (e-cigarettes) and n = 17,831 (cigarettes or other tobacco products). A composite measure of exposure to any tobacco product marketing when watching television or streaming services, or going to the movies (overall) was assessed among respondents categorized as exposed for at least one measure or unexposed across both measures (n = 17,644).

** Assessed by the questions, “When you read newspapers or magazines, how often do you see ads or promotions for (e-cigarettes; cigarettes or other tobacco products)?” Response options for both questions included, “I do not read newspapers or magazines,” “never,” “rarely,” “sometimes,” “most of the time,” and “always.” Respondents were categorized as exposed if they reported “sometimes,” “most of the time,” or “always”; respondents were categorized as unexposed if they reported “never” or “rarely.” Persons who reported “I do not read newspapers or magazines” were set to missing and excluded from the analyses. Total unweighted denominators for exposure to tobacco product marketing included n = 10,209 (e-cigarettes) and n = 10,375 (cigarettes or other tobacco products). A composite measure of exposure to any tobacco product marketing when reading newspapers or magazines (overall) was assessed among respondents categorized as exposed for at least one measure or unexposed across both measures (n = 9,835).

^††^ A composite measure of any advertising or promotion exposure (any source) was assessed based on exposure to retail stores; the Internet, television, streaming services, movies; and newspapers or magazines. Total unweighted denominators included n = 18,534 (e-cigarettes), n = 18,621 (cigarettes or other tobacco products), and n = 18,695 (overall).

^§§^ Estimated weighted total number of users was rounded down to the nearest 10,000 persons.

^¶¶^ Hispanic persons could be of any race.

*** Respondents were instructed as follows: “The next four questions ask about issues related to e-cigarette advertisement. Do not think about cigarettes or other tobacco products.”

^†††^ Respondents were instructed as follows: “The next four questions ask about issues related to advertisements for tobacco products such as cigarettes, cigars, smokeless tobacco, hookahs, roll-your-own cigarettes, pipes, snus, dissolvable tobacco, and bidis. Do not think of electronic cigarettes.”

### Curiosity About and Susceptibility to Tobacco Product Use

Among middle and high school students who were never users of the specific tobacco product, 39.1% were curious about using e-cigarettes, 37.0% were curious about smoking cigarettes, 28.0% were curious about smoking cigars, 23.2% were curious about smoking hookahs, and 15.9% were curious about using smokeless tobacco products (Table 8). Among never users of the specific tobacco product, 45.9% reported susceptibility to cigarettes, followed by e-cigarettes (45.0%), cigars (35.9%), hookahs (29.9%), and smokeless tobacco products (21.2%). Susceptibility to using e-cigarettes was 46.9% among females and 43.4% among males. Susceptibility to using e-cigarettes and smoking cigarettes was higher among middle school students (47.0% and 49.5%, respectively) than among high school students (42.8% and 42.7%, respectively).

**TABLE 8:** Curiosity* about and susceptibility^†^ to tobacco product use among never users of each specific product, by school level, sex, and race/ethnicity — National Youth Tobacco Survey, United States, 2019

Characteristic	Curiosity		Susceptibility	
	% (95% CI)	Estimated no.^§^	% (95% CI)	Estimated no.
**E-cigarettes**				
**Overall**	**39.1 (37.7–40.4)**	**6,820,000**	**45.0 (43.6–46.5)**	**7,820,000**
Sex				
Male	37.3 (35.8–38.8)	3,330,000	43.4 (41.7–45.1)	3,850,000
Female	41.1 (39.2–43.0)	3,450,000	46.9 (44.9–48.8)	3,930,000
Race/Ethnicity				
White, non-Hispanic	39.8 (38.1–41.5)	3,630,000	45.2 (43.4–46.9)	4,110,000
Black, non-Hispanic	32.0 (28.9–35.1)	810,000	38.3 (34.8–41.8)	960,000
Hispanic**^¶^**	42.3 (40.5–44.2)	1,790,000	49.1 (46.9–51.3)	2,060,000
Other, non-Hispanic	39.8 (35.4–44.4)	430,000	46.2 (41.7–50.7)	500,000
School level				
Middle school	40.6 (38.9–42.4)	3,840,000	47.0 (45.1–48.9)	4,420,000
High school	37.2 (34.9–39.6)	2,950,000	42.8 (40.6–44.9)	3,370,000
**Cigarettes**				
**Overall**	**37.0 (35.8–38.2)**	**8,320,000**	**45.9 (44.6–47.3)**	**10,330,000**
Sex				
Male	36.8 (35.5–38.2)	4,180,000	46.4 (44.8–47.9)	5,260,000
Female	37.2 (35.4–39.0)	4,090,000	45.5 (43.7–47.4)	5,010,000
Race/Ethnicity				
White, non-Hispanic	37.2 (35.8–38.6)	4,490,000	45.7 (44.2–47.3)	5,520,000
Black, non-Hispanic	29.8 (26.4–33.3)	910,000	38.4 (34.1–42.9)	1,180,000
Hispanic	40.5 (38.4–42.7)	2,240,000	50.5 (48.3–52.8)	2,780,000
Other, non-Hispanic	38.4 (33.9–43.1)	500,000	46.1 (41.4–50.8)	600,000
School level				
Middle school	39.9 (38.5–41.3)	4,310,000	49.5 (47.7–51.3)	5,350,000
High school	34.3 (32.3–36.4)	3,980,000	42.7 (40.6–44.8)	4,950,000
**Cigars**				
**Overall**	**28.0 (27.0–28.9)**	**6,440,000**	**35.9 (34.9–37.0)**	**8,250,000**
Sex				
Male	31.1 (29.8–32.4)	3,590,000	38.9 (37.6–40.3)	4,480,000
Female	24.8 (23.7–25.9)	2,810,000	32.8 (31.6–34.1)	3,710,000
Race/Ethnicity				
White, non-Hispanic	27.0 (25.8–28.4)	3,410,000	34.6 (33.2–36.1)	4,350,000
Black, non-Hispanic	26.0 (23.9–28.3)	740,000	34.1 (31.6–36.8)	980,000
Hispanic	31.6 (29.7–33.5)	1,790,000	40.8 (38.6–43.0)	2,300,000
Other, non-Hispanic	27.4 (24.2–30.9)	360,000	32.9 (29.6–36.4)	440,000
School level				
Middle school	26.5 (25.3–27.7)	2,930,000	34.6 (33.1–36.1)	3,810,000
High school	29.3 (27.9–30.7)	3,480,000	37.2 (35.7–38.7)	4,400,000
**Smokeless tobacco products**				
**Overall**	**15.9 (14.7–17.1)**	**3,950,000**	**21.2 (19.7–22.8)**	**5,230,000**
Sex				
Male	18.7 (16.9–20.6)	2,300,000	24.6 (22.2–27.3)	3,010,000
Female	13.1 (12.0–14.3)	1,620,000	17.7 (16.5–19.1)	2,180,000
Race/Ethnicity				
White, non-Hispanic	16.8 (15.4–18.3)	2,220,000	21.7 (19.9–23.6)	2,860,000
Black, non-Hispanic	9.8 (8.3–11.6)	330,000	14.4 (12.3–16.7)	480,000
Hispanic	16.6 (15.0–18.3)	1,040,000	23.0 (21.0–25.1)	1,420,000
Other, non-Hispanic	17.5 (14.3–21.2)	240,000	22.3 (18.6–26.5)	300,000
School level				
Middle school	19.2 (17.8–20.7)	2,180,000	25.7 (23.9–27.6)	2,900,000
High school	13.1 (11.6–14.7)	1,750,000	17.3 (15.6–19.2)	2,300,000
**Hookahs**				
**Overall**	**23.2 (22.2–24.2)**	**5,790,000**	**29.9 (28.9–31.0)**	**7,390,000**
Sex				
Male	21.6 (20.2–23.0)	2,780,000	28.5 (27.0–30.0)	3,620,000
Female	25.0 (23.7–26.4)	2,990,000	31.5 (30.0–33.0)	3,730,000
Race/Ethnicity				
White, non-Hispanic	21.7 (20.5–23.1)	3,020,000	27.9 (26.5–29.3)	3,860,000
Black, non-Hispanic	22.4 (20.3–24.6)	700,000	30.1 (28.1–32.3)	920,000
Hispanic	28.1 (26.3–30.0)	1,670,000	35.5 (33.5–37.6)	2,100,000
Other, non-Hispanic	20.7 (18.1–23.6)	280,000	25.9 (23.0–29.0)	340,000
School level				
Middle school	18.8 (17.5–20.1)	2,130,000	25.2 (23.7–26.8)	2,840,000
High school	26.9 (25.5–28.4)	3,630,000	33.8 (32.3–35.4)	4,520,000

**Abbreviations:** CI = confidence interval; e-cigarettes = electronic cigarettes.

* Assessed by the question, “Have you ever been curious about (tobacco product)?” Responses were recoded as highly curious (definitely yes, probably yes, or probably not) and not curious (definitely not). Overall estimates of curiosity were calculated among never tobacco product users of the specific tobacco product: e-cigarettes (n = 12,523), cigarettes (n = 16,031), cigars (n = 16,411), smokeless tobacco (chewing tobacco, snuff, and dip; n = 17,664), and hookahs (n = 17,627).

^†^ Assessed by the questions, “Do you think that you will use (tobacco product) soon? Do you think you will use (tobacco product) in the next year? If one of your best friends were to offer you (tobacco product), would you try it? Have you ever been curious about (tobacco product)?” Susceptibility was defined as a response other than “definitely not” to any of the four questions. Respondents with a missing value for all four questions or any combination of “definitely not” and missing value or values were recoded as missing for susceptibility. Overall estimates of susceptibility were calculated among never tobacco product users of the specific tobacco product: e-cigarettes (n = 12,448), cigarettes (n = 16,010), cigars (n = 16,347), smokeless tobacco (chewing tobacco, snuff, and dip; n = 17,513), and hookahs (n = 17,549).

^§^ Estimated weighted total numbers were rounded down to the nearest 10,000 persons. Overall estimates might not directly total to sums of corresponding subgroup estimates because of rounding or inclusion of students who did not self-report sex, race/ethnicity, or grade level.

^¶^ Hispanic persons could be of any race.

### Harm Perceptions

**FIGURE 3:**
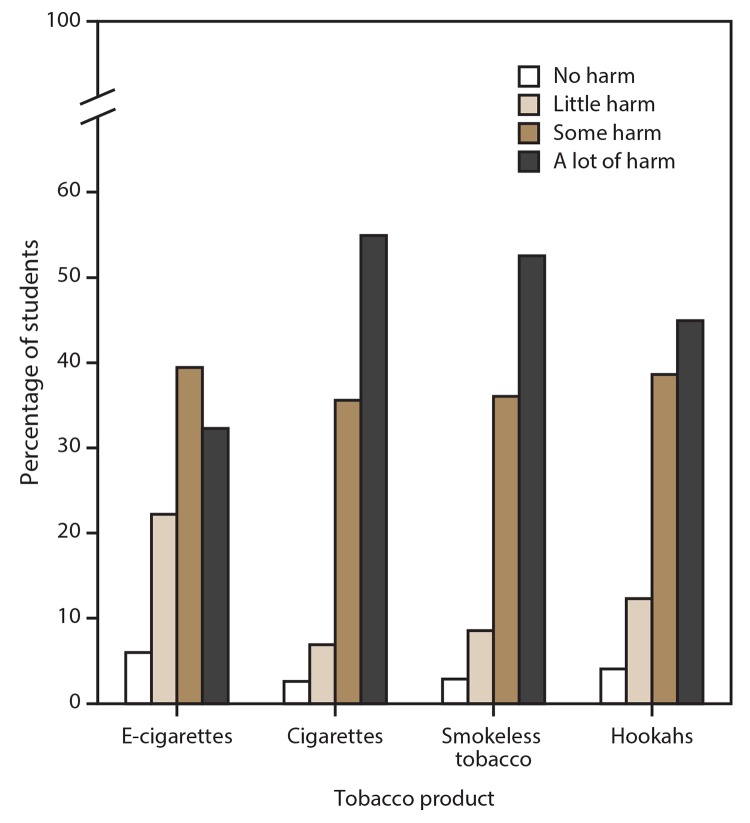
Harm perceptions of intermittent use of e-cigarettes, cigarettes, smokeless tobacco, and hookahs* reported by middle and high school students — National Youth Tobacco Survey, United States, 2019 **Abbreviation:** E-cigarettes = electronic cigarettes. * Assessed by the questions, “How much do you think people harm themselves when they (smoke cigarettes; use chewing tobacco, snuff, dip, snus, or dissolvable tobacco products; use e-cigarettes; or smoke tobacco in a hookah or water pipe) some days but not every day?” Response options included “no harm,” “little harm,” “some harm,” and “a lot of harm” for each of the four tobacco products assessed. Harm perceptions of intermittent use of other tobacco products were not assessed in the 2019 National Youth Tobacco Survey.

The percentage of middle school and high school students who reported that intermittent use of tobacco products causes a lot of harm was highest for cigarettes (54.9%), followed by smokeless tobacco products (52.5%), hookahs (44.9%), and e-cigarettes (32.3%) (Figure 3). The percentage of students who reported that intermittent use causes no or little harm was highest for e-cigarettes (28.2%), followed by hookahs (16.4%), smokeless tobacco products (11.5%), and cigarettes (9.5%).

### Urges to Use Tobacco Products

Among current users of any tobacco product, 24.7% reported experiencing cravings to use tobacco products during the past 30 days, including 25.8% of high school students and 21.4% of middle school students (Table 9). The prevalence of experiencing cravings was reported by 28.7% of non-Hispanic whites, 18.3% of Hispanics, and 15.8% of non-Hispanic blacks. Overall, 13.7% of current tobacco product users reported wanting to use a tobacco product within 30 minutes of waking, including 15.6% of high school students and 7.3% of middle school students. The prevalence of wanting to use a tobacco product within 30 minutes of waking was reported by 16.5% of non-Hispanic white and 9.2% of Hispanic students.

**TABLE 9:** Urges to use tobacco products and quitting behaviors among middle and high school students who reported current tobacco product use,* by school level, sex, and race/ethnicity — National Youth Tobacco Survey, United States, 2019

Characteristic	Urges to use tobacco products				Quitting behaviors			
	Past 30-day craving^†^		Within 30 minutes of waking^§^		Thinking about quitting^¶^		Past-year quit attempt**	
	% (95% CI)	Estimated no.^††^	% (95% CI)	Estimated no.	% (95% CI)	Estimated no.	% (95% CI)	Estimated no.
**Overall**	**24.7 (22.0–27.6)**	**1,510,000**	**13.7 (11.7–16.0)**	**830,000**	**57.8 (55.5–60.0)**	**3,330,000**	**57.5 (55.4–59.6)**	**3,300,000**
Sex								
Male	23.8 (19.9–28.1)	760,000	15.7 (12.7–19.4)	500,000	56.8 (54.2–59.2)	1,740,000	57.0 (54.3–59.6)	1,730,000
Female	25.8 (22.9–28.9)	740,000	11.5 (9.5–13.9)	330,000	58.9 (55.6–62.1)	1,580,000	58.0 (55.0–61.0)	1,560,000
Race/Ethnicity								
White, non-Hispanic	28.7 (25.5–32.1)	1,070,000	16.5 (13.9–19.5)	610,000	56.2 (54.2–58.9)	1,990,000	55.3 (52.4–58.2)	1,950,000
Black, non-Hispanic	15.8 (12.0–20.6)	100,000	—**^§§^**	—	59.5 (53.8–65.0)	370,000	59.1 (53.3–64.6)	360,000
Hispanic^¶¶^	18.3 (15.1–21.9)	250,000	9.2 (7.2–11.6)	120,000	61.6 (58.0–65.0)	810,000	62.9 (59.0–66.7)	820,000
Other, non-Hispanic	—	—	—	—	56.7 (45.8–67.1)	120,000	56.9 (48.6–64.9)	120,000
**High school**	**25.8 (22.6–29.4)**	**1,200,000**	**15.6 (13.2–18.4)**	**720,000**	**57.7 (55.3–60.0)**	**2,540,000**	**55.7 (53.2–58.2)**	**2,440,000**
Sex								
Male	25.5 (21.1–30.5)	630,000	18.0 (14.4–22.2)	440,000	57.2 (54.3–60.0)	1,350,000	56.0 (53.1–58.9)	1,310,000
Female	26.3 (22.9–30.0)	560,000	13.0 (10.7–15.8)	280,000	58.2 (54.8–61.5)	1,180,000	55.3 (51.7–58.8)	1,120,000
Race/Ethnicity								
White, non-Hispanic	29.9 (26.2–33.8)	900,000	18.7 (15.8–22.1)	560,000	56.2 (53.1–59.3)	1,620,000	53.8 (50.4–57.1)	1,540,000
Black, non-Hispanic	16.1 (11.7–21.8)	70,000	—	—	61.2 (56.1–66.2)	280,000	58.7 (52.4–64.6)	260,000
Hispanic	17.4 (14.0–21.4)	160,000	9.4 (6.8–12.9)	80,000	61.6 (57.2–65.7)	530,000	60.3 (55.2–65.1)	520,000
Other, non-Hispanic	—	—	—	—	52.7 (41.6–63.5)	80,000	56.8 (48.3–64.9)	90,000
**Middle school**	**21.4 (18.1–25.1)**	**310,000**	**7.3 (5.8–9.2)**	**100,000**	**57.9 (52.3–63.4)**	**770,000**	**63.3 (59.3–67.1)**	**840,000**
Sex								
Male	18.2 (14.7–22.4)	130,000	—	—	54.9 (48.3–61.5)	370,000	59.6 (53.6–65.4)	400,000
Female	24.6 (19.4–30.8)	170,000	—	—	61.0 (54.3–67.3)	390,000	66.7 (61.4–71.5)	430,000
Race/Ethnicity								
White, non-Hispanic	23.7 (18.5–29.9)	160,000	—	—	55.8 (49.3–62.1)	360,000	62.4 (54.7–69.5)	410,000
Black, non-Hispanic	—	—	—	—	54.6 (41.4–67.2)	90,000	60.3 (51.5–68.5)	90,000
Hispanic	20.3 (15.7–25.8)	90,000	—	—	61.7 (54.9–68.0)	260,000	67.6 (61.7–72.9)	280,000
Other, non-Hispanic	—	—	—	—	—	—	—	—

**Abbreviation:** CI = confidence interval.

* Any current tobacco product use was defined as use of any tobacco product (e-cigarettes, cigarettes, cigars, smokeless tobacco, hookahs, pipe tobacco, or bidis [small brown cigarettes wrapped in a leaf]) on ≥1 day during the past 30 days (n = 4,198).

^†^ Assessed by the question, “During the past 30 days, have you had a strong craving or felt like you really needed to use a tobacco product of any kind?” The response options were yes or no.

^§^ Assessed by the question, “How soon after you wake up do you want to use a tobacco product?” Response options were dichotomized as within 30 minutes (within 5 minutes or from 6 to 30 minutes) or not within 30 minutes (from more than 30 minutes to 1 hour, after >1 hour but <24 hours, “I rarely want to use tobacco products,” or “I do not want to use tobacco products”).

^¶^ Assessed by the question, “Are you seriously thinking about quitting the use of all tobacco products?” Response options were dichotomized as yes (“yes, during the next 30 days”; “yes, during the next 6 months”; “yes, during the next 12 months”; or “yes, but not during the next 12 months”) or no (“no, I am not thinking about quitting the use of all tobacco products”).

** Assessed by the question, “During the past 12 months, how many times have you stopped using all tobacco products for one day or longer because you were trying to quit all tobacco products for good?” Response options were dichotomized as yes (one time, two times, three to five times, six to nine times, or ≥10 times) or no (“no, I did not try to quit during the past 12 months”).

^††^ Estimated weighted total numbers of users were rounded down to the nearest 10,000 persons. Overall estimates might not directly total to sums of corresponding subgroup estimates because of rounding or inclusion of students who did not self-report sex, race/ethnicity, or grade level.

^§§^ Data were statistically unreliable because of unweighted denominator <50 or a relative standard error >30%.

^¶¶^ Hispanic persons could be of any race.

### Quitting Behaviors

Among current users of any tobacco product, 57.8% reported they were seriously thinking about quitting the use of all tobacco products (Table 9). By school level, 57.7% of high school student current users and 57.9% of middle school student current users reported they were seriously thinking about quitting. Furthermore, 57.5% of current tobacco product users reported they stopped using all tobacco products for ≥1 day because they were trying to quit, including 55.7% of high school students and 63.3% of middle school students.

## Discussion

### Public Health Implications

Findings from the 2019 NYTS indicate that approximately two in five students (40.5%), including approximately half of high school students (53.3%) and one in four middle school students (24.3%), had ever tried a tobacco product. Furthermore, approximately one in four students (23.0%), including approximately three in 10 high school students (31.2%) and one in eight middle school students (12.5%), had used a tobacco product during the past 30 days. Approximately one in three current tobacco product users (33.9%) reported using multiple tobacco products; youths who use multiple tobacco products are at higher risk for developing nicotine dependence and might be more likely to continue using tobacco into adulthood (*1*,*2*). Although most current youth tobacco product users are not daily users (*9*,*12*,*13*), estimates of frequent e-cigarette use among high school students were comparable to those observed for cigarette and smokeless tobacco product users in 2019. However, even infrequent tobacco product use (1–5 days during the past 30 days) can lead to symptoms of nicotine dependence (*11*); in 2019, among current tobacco product users, approximately one in four high school students and one in five middle school students reported having cravings for tobacco products. Youth use of tobacco products in any form is unsafe, regardless of whether the products are smoked, smokeless, or electronic (*2*,*3*). Continued efforts are warranted to prevent and reduce all forms of tobacco product use among U.S. youths.

In 2019, the prevalence of cigarette smoking among youths was the lowest ever captured by the NYTS since 1999. An estimated 5.8% of high school students and 2.3% of middle school students reported current cigarette smoking in 2019, compared with 28.5% of high school students and 9.2% of middle school students in 1999 (*14*). However, youths are using various other tobacco products, most notably e-cigarettes.

Since 2014, e-cigarettes have remained the most commonly used tobacco product among U.S. youths (*15*,*16*). During 2017–2018, current e-cigarette use increased by 77.8% among high school students and 48.5% among middle school students (*16*,*17*). The 2017–2018 surge in e-cigarette use prompted the U.S. Surgeon General to issue an advisory declaring e-cigarette use among youths an epidemic in December 2018. This advisory underscored the importance of protecting youths from a lifetime of nicotine dependency and associated health risks (*18*). In 2019, approximately three in 11 high school students (27.5%) and one in 10 middle school students (10.5%) used e-cigarettes during the past 30 days, which is higher than estimates observed in the 2018 NYTS (*16*,*17*), However, direct attribution of this change to actual increases in product use is not possible because changes made to the 2019 survey could also lead to higher estimates of use. Changes included the electronic mode of survey administration, preamble descriptions (e.g., specific brand examples), and tobacco product images.*

The 2019 NYTS offers insights into factors known to promote tobacco product use among youths. For example, tobacco marketing can prompt tobacco product initiation and use among youths (*2*,*3*,*19*). Flavors in tobacco products can increase the appeal of tobacco products to youths (*2*,*3*). Measuring curiosity and susceptibility can help identify youths who might progress from nonuse to experimentation and further progression to established tobacco product use (*20*–*23*). Although the 2009 Family Smoking Prevention and Tobacco Control Act^†^ prohibits characterizing flavors other than tobacco and menthol in cigarettes, characterizing flavors in other tobacco products, such as e-cigarettes, are widely available (*24*,*25*). Efforts to address these factors, including strategies to curb e-cigarette marketing that is appealing to young persons and strategies to reduce access to flavored tobacco products by young persons, could help prevent and reduce tobacco product use by youths (*18*). In March 2019, FDA published a draft guidance proposing to end the previous compliance policy as it applied to flavored e-cigarettes and other electronic nicotine delivery systems (ENDS) and prioritizing enforcement of flavored ENDS products, including warning letters and civil money penalties for ENDS that do not have a marketing authorization from FDA (*26*).

Because of the especially high prevalence of e-cigarette use among U.S. youths, increasing successful quit attempts could complement prevention efforts to reduce tobacco product use among youths. In 2019, approximately half of middle and high school current tobacco product users reported seriously thinking about quitting all tobacco products or making a past-year quit attempt. The U.S. Preventive Services Task Force recommends that primary care clinicians provide interventions, including education or brief counseling, to prevent initiation of tobacco use among school-aged children and adolescents (*27*). Additional tailored interventions and services could further support cessation of all tobacco product use among youths.

### Public Health Action

The sustained implementation of population-based strategies, along with regulation of tobacco products by FDA, is critical to preventing and reducing all forms of tobacco product use among youths (*1*–*3*,*18*). Strategies to reduce tobacco product use and initiation among youths include increasing prices of tobacco products; protecting persons from exposure to secondhand smoke and e-cigarette aerosol; sustaining hard-hitting media campaigns that warn about the dangers of tobacco product use; restricting youth access to tobacco products, including increasing the minimum age for purchase of tobacco products to 21 years; and prohibiting the sale of flavored tobacco products (*1*,*16*,*18*,*28*).

Everyone can help protect youths from the harms of tobacco products, including e-cigarettes (*3*,*18*). Parents and educators can learn about the different types of e-cigarettes available, including discreet devices shaped like USB flash drives (e.g., JUUL). They and others who influence youths can set a positive example by being tobacco free and communicating that nicotine use can lead to addiction and can harm the developing brain and affect learning, memory, and attention (*3*). Schools can adopt and enforce tobacco-free campus policies that include e-cigarettes and reject tobacco industry–sponsored prevention programs. Of the tobacco industry–sponsored prevention programs that have been studied, none have been shown to be effective (*2*). Furthermore, health care providers can ask about the use of all tobacco products, including e-cigarettes, when screening patients for tobacco product use and assisting those who want to quit using tobacco products.

## Limitations

The findings in this report are subject to at least three limitations. First, because NYTS transitioned survey administration modes from a paper and pencil survey to an electronic survey in 2019, statistical tests comparing measures with previous years were not done. Second, data were self-reported and might be subject to recall and response bias. However, the validity of self-reported tobacco product use is consistently high in population-based studies (*29*,*30*). Finally, data were collected only from middle and high school students who attended public or private schools; findings might not be generalizable to youths who are home schooled, have dropped out of school, are in detention centers, or are enrolled in alternative schools. However, data from the Current Population Survey indicate that approximately 97% of U.S. youths aged 10–17 years were enrolled in a traditional school in 2017 (*31*).

## Conclusion

NYTS is the only comprehensive, nationally representative survey of U.S. middle and high school students focused on tobacco product use behaviors and associated factors. Findings from NYTS indicate that in 2019, approximately half of high school students (53.3%) and one in four middle school students (24.3%) had ever used a tobacco product. Furthermore, approximately three in 10 high school students (31.2%) and approximately one in eight middle school students (12.5%) had used a tobacco product during the past 30 days. Multiple factors known to promote tobacco product use and initiation among youths (*2*,*3*), including flavored tobacco products, marketing, curiosity and susceptibility, and misperceptions of harm, remained prevalent. The comprehensive and sustained implementation of evidence-based tobacco control strategies, combined with FDA’s regulation of tobacco products, is important for preventing and reducing all forms of tobacco product use among U.S. youths. In addition, because tobacco products might continue to diversify, surveillance among youths for all forms of tobacco product use and associated factors is important to the development of public health policy and action at the national, state, and community levels.

## Footnotes

* The 2018 NYTS questionnaire provided the following descriptive preamble for e-cigarettes: “The next 11 questions are about electronic cigarettes or e-cigarettes. E-cigarettes are battery powered devices that usually contain a nicotine-based liquid that is vaporized and inhaled. You may also know them as e-cigs, vape-pens, hookah-pens, e-hookahs, e-cigars, e-pipes, personal vaporizers, or mods. Some brand examples include NJOY, Blu, Vuse, MarkTen, Logic, Vapin Plus, eGo, and Halo.” The 2019 NYTS questionnaire provided the following descriptive preamble for e-cigarettes: “The next several questions are about electronic cigarettes or e-cigarettes. Some brand examples include JUUL, Vuse, MarkTen, and blu. E-cigarettes are battery powered devices that usually contain a nicotine-based liquid that is vaporized and inhaled. You may also know them as e-cigs, vape-pens, e-hookahs, vapes, or mods.” In the 2019 questionnaire, preambles for each tobacco product section, including e-cigarettes, contained images showing examples of each respective product.

† Family Smoking Prevention and Tobacco Control Act, Pub. L. No. 111-31, H.R. 1256 (June 22, 2009).
